# The first direct detection of spotted fever group *Rickettsia* spp. diversity in ticks from Ningxia, northwestern China

**DOI:** 10.1371/journal.pntd.0012729

**Published:** 2025-01-02

**Authors:** Wen-Jie Zhu, Run-Ze Ye, Di Tian, Ning Wang, Wan-Ying Gao, Bai-Hui Wang, Zhe-Tao Lin, Ya-Ting Liu, Yi-Fei Wang, Dai-Yun Zhu, Yi Sun, Xiao-Yu Shi, Wen-Qiang Shi, Na Jia, Jia-Fu Jiang, Xiao-Ming Cui, Zhi-Hong Liu, Wu-Chun Cao

**Affiliations:** 1 State Key Laboratory of Pathogen and Biosecurity, Academy of Military Medical Sciences, Beijing, P.R. China; 2 Department of Emergency Medicine, Qilu Hospital of Shandong University, Jinan, Shandong, P.R. China; 3 School of Public Health, Ningxia Medical University, Yinchuan, Ningxia, P.R. China; 4 Institute of EcoHealth, School of Public Health, Shandong University, Jinan, Shandong, P.R. China; Postgraduate Institute of Medical Education and Research, INDIA

## Abstract

**Background:**

Tick-borne infectious diseases caused by the spotted fever group *Rickettsia* (SFGR) have continuously emerging, with many previously unidentified SFGR species reported. The prevalence of SFGRs in northwestern China remains unclear. This study aimed to examine the prevalence of SFGRs and *Anaplasma* species by analyzing tick samples collected from the Ningxia region.

**Methods:**

During 2022–2023, ticks were collected from Ningxia, northwestern China, and screened using PCR to amplify target genes (16S rRNA, *gltA*, *ompA* and *groEL*). The amplicons were confirmed by Sanger sequencing. Single-gene sequences and concatenated sequences were used to infer phylogenetic relationships for identifying *Rickettsia* species.

**Results:**

Out of the 425 DNA samples, a total of 210 samples tested positive for SFGRs in ticks from Ningxia, China, with a relatively high positive rate of 49.4% (210/425). Eight spotted fever group rickettsiae and one *Anaplasma* species were identified and characterized, including *Rickettsia raoultii* (102, 24.0%), *R*. *aeschlimannii* (65, 15.3%), *R*. *sibirica* (12, 2.8%), *R*. *slovaca* (4, 0.9%), *R*. *heilongjiangensis* (1, 0.2%), *Cadidatus* Rickettsia hongyuanensis (4, 0.9%), *Ca*. R. jingxinensis (11, 2.6%), *Ca*. R. vulgarisii (11, 2.6%) and *Anaplasma ovis* (98, 23.1%). The positive rate of bacterial species ranged from 0.2% to 24.0%. Interestingly, one novel *Rickettsia* species, provisionally named “*Candidatus* Rickettsia vulgarisii”, was detected in *Argas* ticks from Zhongwei city, which suggests the possibility of local transmission to other areas through birds. Genetic and phylogenetic analysis based on the 16S rRNA, *gltA*, *ompA*, and *17kDa* genes indicated that it was divergent from all known SFG *Rickettsia* species but mostly related to *R*. *vini*. Different SFGR species were associated with specific tick species or genera. In addition, *Anaplasma ovi*s was detected in two *Dermacentor* species, and co-infection with SFGRs was observed in 14.6% (62/425) of samples.

**Conclusions:**

This study describes the prevalence and diversity of SFGRs in ticks from Ningxia for the first time by direct detection, reveals that *Rickettsia* diversity related to tick species. This data suggests that surveillance for tick-borne SFGR infections among human populations should be enhanced in this region, and further investigations on their pathogenicity to humans and domestic animals are still needed.

## Author summary

SFGRs are bacteria mainly vectored by different tick species. There is increasing attention paid to them as causative agents of human disease. To date, these bacteria have been prevailing in China, but no studies have been conducted to detect potential tick vectors of rickettsiae in the Ningxia region. This study collected a total of 425 ticks from four cities with diverse geographic environments in Ningxia and determined the presence of SFGRs and *Anaplasma* spp. in naturally infected ticks. All the ticks were classfied as *Argas*, *Dermacentor*, *Haemaphysalis* s and *Hyalomma* genera. The overall prevalence of SFGRs and *Anaplasma* spp. detected in nine tick species were 49.4% and 23.1%, respectively. We present data that indicate high genetic diversity of SFGRs in Ningxia and co-infections with SFGR and *A*. *ovis* in ticks. Importantly, we describe a divergent *Rickettsia* spp. and propose the name *Candidatus* Rickettsia vulgarisii. These findings imply a potential threat of SFGRs infection among humans and highlight the importance of SFGRs and their targeted surveillance for human health in Ningxia.

## Introduction

Ticks are considered important arthropod vectors of various pathogens and have been associated with serious medical and veterinary health problems [1]. Many tick-borne bacterial agents are significant causes of unknown morbidity and mortality in human and domestic animals of great public health importance, e.g., spotted fever group rickettsiae (SFGRs), *Anaplasma* spp. and *Coxiella* spp. [2,3]. The majority of tick-borne infections are zoonotic, and their incidence and distribution are steadily increasing worldwide [4,5]. The distribution of various tick species and tick-borne agents in China has been studied for a long time. To date, at least 124 tick species from 9 genera have been reported across 1134 counties in China [6]. It was reported that more than 3,500 human cases had been confirmed infections with tick-borne pathogens (*Borrelia* spp., *Anaplasma* spp., *Babesia* spp. and SFGRs) covering the west, north, and northeast of China, including 32 cases of unexplained fever caused by Rickettsioses [6,7].

*Rickettsia* spp. and *Anaplasma* spp. belonging to the order Rickettsiales, of most concern in China, are common tick-borne bacterial pathogens. The SFGRs are obligate intracellular, gram-negative bacteria, generally associated with ticks, which can cause the emergence of spotted fever worldwide [8–11]. More than 20 species of SFGRs are causative agents of human diseases characterized by various clinical features, including fever, headache, rash, and cervical lymphadenopathy, which can be fatal in severe cases [12–15]. In mainland China, the emergence of SFGRs and corresponding rickettsial human cases have been predominantly reported in northeastern and central regions, where climatic conditions and human activities such as farming and livestock rearing favor the proliferation of tick populations, whereas in other areas, rickettsiosis is sporadic [16–20]. Several cases of rickettsial disease associated with acute fever and lymphadenopathy have been reported in Henan and Xinjiang regions of China [19,20]. In addition to humans, SFGRs infections have been detected in a variety of animal reservoir, including artiodactyla animals (sheep/goat/horse) [21,22], rodents [2], and wild birds [23]. Meanwhile, it is important to note that significant differences exist among tick species and their carried rickettsiae [24,25]. *Anaplasma* species have been also frequently detected in ticks and animals from multiple provinces in China [26]. Domestic animals, including sheep, cattle and goats, are often infected causing weight loss, reduced milk production, or even death, leading to great economic losses in animal husbandry annually [27]. Human infectious agents with these pathogens, such as *A*. *phagocytophilum*, *A*. *capra* and *A*. *bovis*, have been found in Inner Mongolia, Heilongjiang and Anhui provinces of China, but not as often as rickettsial cases [2,28–31].

Diverse manifestations of diseases can make their clinical diagnoses rather difficult. With the aid of molecular techniques, recent studies have expanded our knowledge on the diversity of vetor-tick bacteria and many novel species are being discovered globally with increasing frequency [31,32]. Some tick-borne bacteria species that were previously not considered pathogenic to humans are nowadays proven pathogens [33]. Diagnosis of infections with rickettsiae is commonly achieved by employing molecular biology-based analyses, specifically polymerase chain reaction (PCR) and nucleotide sequencing of DNA extracted from the patient [31,33]. Many different genes have been used for *Rickettsia* and *Anaplasma* phylogenetic systematics, including 16S rRNA, *gltA*, *17kDa*, *ompA*, *sca4*, *groEL*, *msp2* and complete genomic sequences by conventional, nested, and real-time PCR techniques [34]. However, relatively few investigations of tick-borne agents have been reported in the underdeveloped regions of northwestern China [35]. Thus, effective surveillance helps to determine tick populations, pathogen presence and seasonal activity, which is critical to implementing control measures. To be specific, investigations on the presence of tick-borne bacteria circulating in our environment within ticks—are of great medical and public health significance.

Located in northwestern China, Ningxia covers an area of 66,400 km^2^. The large topographic drop and the varied landform make for local complex ecological landscapes and diverse vegetation types. Combined with extensive livestock production activities, which is the major source of income for rural households, these conditions create a favorable environmental for tick growth and development [36,37]. Meanwhile, the frequent contact between villagers and domestic animals makes it possible for tick-borne pathogens and related diseases to be easily transmitted from animals or ticks to humans. The abundance of tick species varies substantially across diverse biogeographic zones defined by climatic and ecological characteristics. This study focuses on Ningxia, a region with distinct ecological and socio-economic characteristics that may promote tick development and tick-borne pathogen transmission. However, data about the prevalence and diversity on tick-borne bacteria in this area are limited, and relevant case studies are equally lacking. There have been no reports of SFGRs and *Anaplasma* species or even other pathogens directly detected from ticks. In this study, we collected ticks from five locations in Ningxia and analyzed the presence, prevalence, and genetic characteristics of the SFGRs and *Anaplasma* spp. in ticks using PCR and multi-locus sequence typing (MLST). Nucleotide sequence analysis and phylogenetic relationships are helpful to pathogen identification. Here we report the first finding of ticks, harbouring the pathogen SFGRs and *Anaplasma* spp. in Ningxia. The results of this study will be valuable in creating effective control measures to prevent zoonotic pathogens from spreading in this underexplored region.

## Methods

### Ethics statement

The collection of ticks from the body surface of host animals in this study was verbally consented by the animal owners and approved by the Animal Experiment Committee of the Laboratory Animal Center, Academy of Military Medical Sciences, China. The animal ethics approval number is IACUC-DWZX-027-20.

### Sample collection and DNA extraction

Ticks were collected from livestock by using forceps, and vegetation surrounding the farms or living areas of animals by dragging white flags between March and May of the tick active period in 2022–2023 in all cities of Ningxia (38°27’58.9”N, 106°16’41.4”E), including Guyuan, Shizuishan, Wuzhong,‌ Yinchuan, ‌and ‌Zhongwei city. Only adult ticks were identified and classified based on morphological criteria by an entomologist (Y.S.). Ticks were frozen and stored at −80°C until DNA extraction individually. We employed ArcGIS v10.8.2 to create detailed maps illustrating the geographical distribution of tick species across Ningxia region. The basemap shapefiles were downloaded from the Chinese Resource and Environmental Science Data Platform (http://www.resdc.cn/, DOI:10.12078/2023010102). These visualizations provide a clear spatial context for the prevalence of different tick species [38]. DNA extraction, amplification, and PCR product detection were carried out in separate rooms in order to prevent cross-contamination. Ticks were washed in distilled water for 10 min dried on sterile filter paper and homogenized individually with a single tick in Eppendorf tubes. Following the manufacturer’s instructions, the TaKaRa RNA/DNA Extraction Kit (TaKaRa, Dalian, China) was used for DNA extraction from homogenized ticks. Obtained total DNA was stored at –80°C.

### PCR assays and sequencing

Ticks were examined for the presence of SFGRs and *Anaplasma* spp. by qualitative PCR (semi-nested and nested PCR) which amplify fragments of the 16S rRNA (*rrs*), outer membrane protein A gene (*ompA*), citrate synthase (*gltA*), and heat shock protein (*groEL*) genes [39,40]. Additionally, *17kDa* gene was recovered for the putative novel *Rickettsia* species by nested PCR. PCR conditions comprised initial denaturation at 94°C for 3 min followed by 35 cycles of denaturation at 94°C for 30 sec, annealing at temperatures, specified in S1 Table, for 30 sec and elongation at 72°C for 1.5 min. PCR primer sequences and conditions are listed in S1 Table. The DNA of *R*. *raoultii* and *A*. *ovis* were used for as positive control, whereas ddH_2_O was set as the negative control. PCR reactions were performed using a Veriti 96-Well Thermal Cycler (Applied Biosystems, Waltham, USA) and the PCR amplicons were subjected to Sanger sequencing in both directions after showing high intensity bands in 1.5% agarose gel electrophoresis. All the obtained nucleotide sequences were proofread, edited and assembled by CLC Main Workbench 5.0 (Qiagen, Redwood City, CA, USA)

### Phylogenetic analysis

Samples that tested positive for all three genes of SFGRs or *Anaplasma* spp. were considered positive. Individual *rrs*, *gltA*, *ompA* or *groEL* sequence of the PCR products was compared to the sequences in the GenBank using the nucleotide Basic Local Alignment Search Tool (BLAST) [41]. Individual gene sequence and concatenated sequences were used for phylogenetic analysis. The assembled gene sequences were concatenated in the order of *rrs*, *ompA* (for *Rickettsia*)/*groEL* (for *Anaplasma*), and *gltA*. Additionally, reference sequences of different genes from various strains were obtained from GenBank, from which the amplified regions were extracted and concatenated in order (S2 Table). These sequences were aligned using MAFFT v7.505 [42] and adjusted using trimAl software [43]. All phylogenetic trees were constructed with maximum likelihood (ML) in IQ-TREE v2.2.0.3 with 1000 bootstrap replicates [44]. To further validate the evolutionary positions of gene sequences in newly discovered *Rickettsia* species, separate phylogenetic trees were constructed for the *rrs*, *ompA*, and *gltA* genes of *Rickettsia* species. These concatenated trees were annotated and visualized using the Tree Visualization by One Table online software [45].

### Statistical analyses

The data obtained in this study were analyzed to estimate the proportion or percentage of SFGRs in different tick species with a 95% confidence interval (95% CI) including continuity correction based on one tick for a sample. Pearson’s chi-square (χ^2^) test or Fisher’s exact test was used to examine the differences in positive rates among tick species. Statistical significance was determined using GraphPad Prism 8 (GraphPad Software Inc., San Diego, California, USA); a *P*-value less than 0.05 was considered to indicate statistical significance. We used R, an open-source statistical programming platform, and the circlize package v0.4.16 to create chord diagrams to visualize the associations between tick species and the prevalence of *Rickettsia* species [46].

## Results

### Morphological identification of ticks

**Fig 1:**
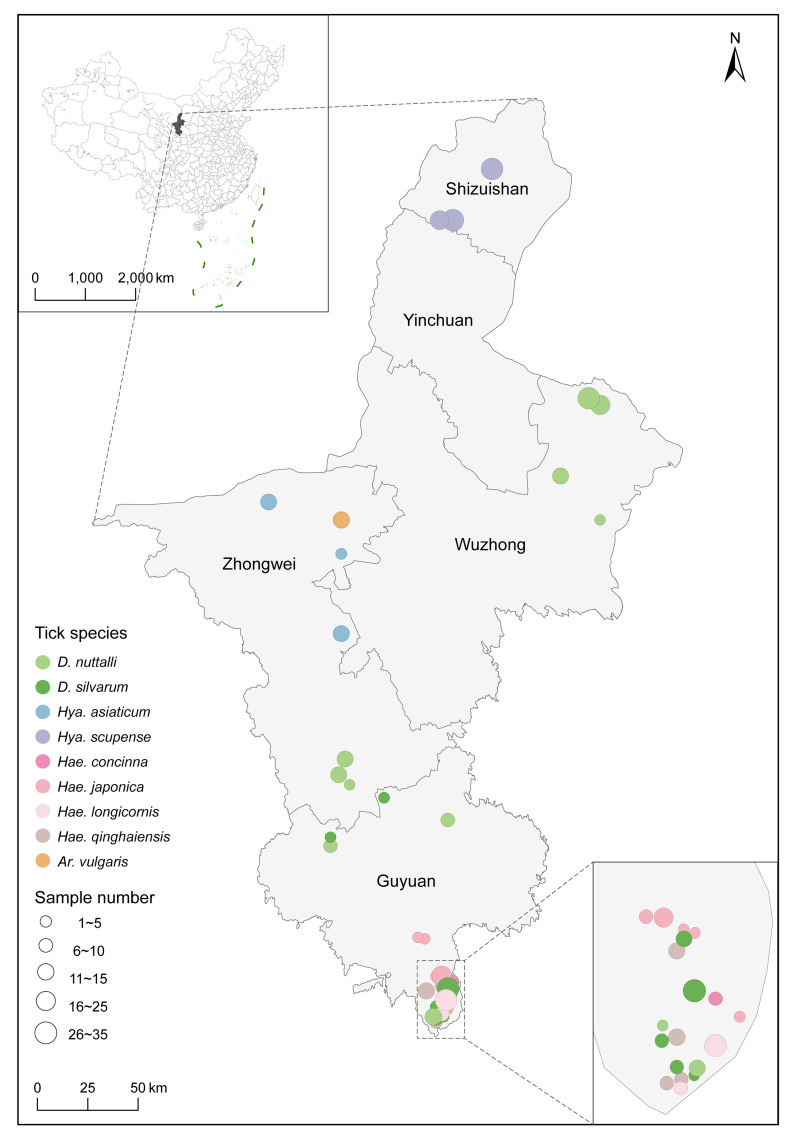
Distribution of tick samples collected in Ningxia, China. Different colour and size of circles represent the species and number of ticks collected from Ningxia, China. The map was constructed using ArcGIS v10.8.2 software. The basemap shapefiles were downloaded from the Chinese Resource and Environmental Science Data Platform (http://www.resdc.cn/, DOI:10.12078/2023010102).

A total of 425 adult ticks were collected in four cities from Ningxia.After morphological identification, the ticks were classified into 4 genera and 9 species. These included *Argas vulgaris* (14, 3.29%), *Dermacentor nuttalli* (121 28.47%), *Dermacentor silvarum* (65, 15.29%), *Haemaphysalis concinna* (10, 2.35%), *Haemaphysalis japonica* (36, 8.47%), *Haemaphysalis longicornis* (42/425, 9.88%), *Haemaphysalis qinghaiensis* (36, 8.47%), *Hyalomma asiaticum* (24, 5.65%), and *Hyalomma scupense* (77, 18.12%). The distribution of tick species varies in different regions of Ningxia. (Fig 1). Ticks were collected in each city except Yinchuan (S3 Table). *Dermacentor* ticks (186/425, 43.8%) were the majority of ticks sampled and notably widespread, spanning the eastern, central, and southern regions of Ningxia (S1 Fig).

#### Detection and characterization of *Rickettsia* spp

**Fig 2:**
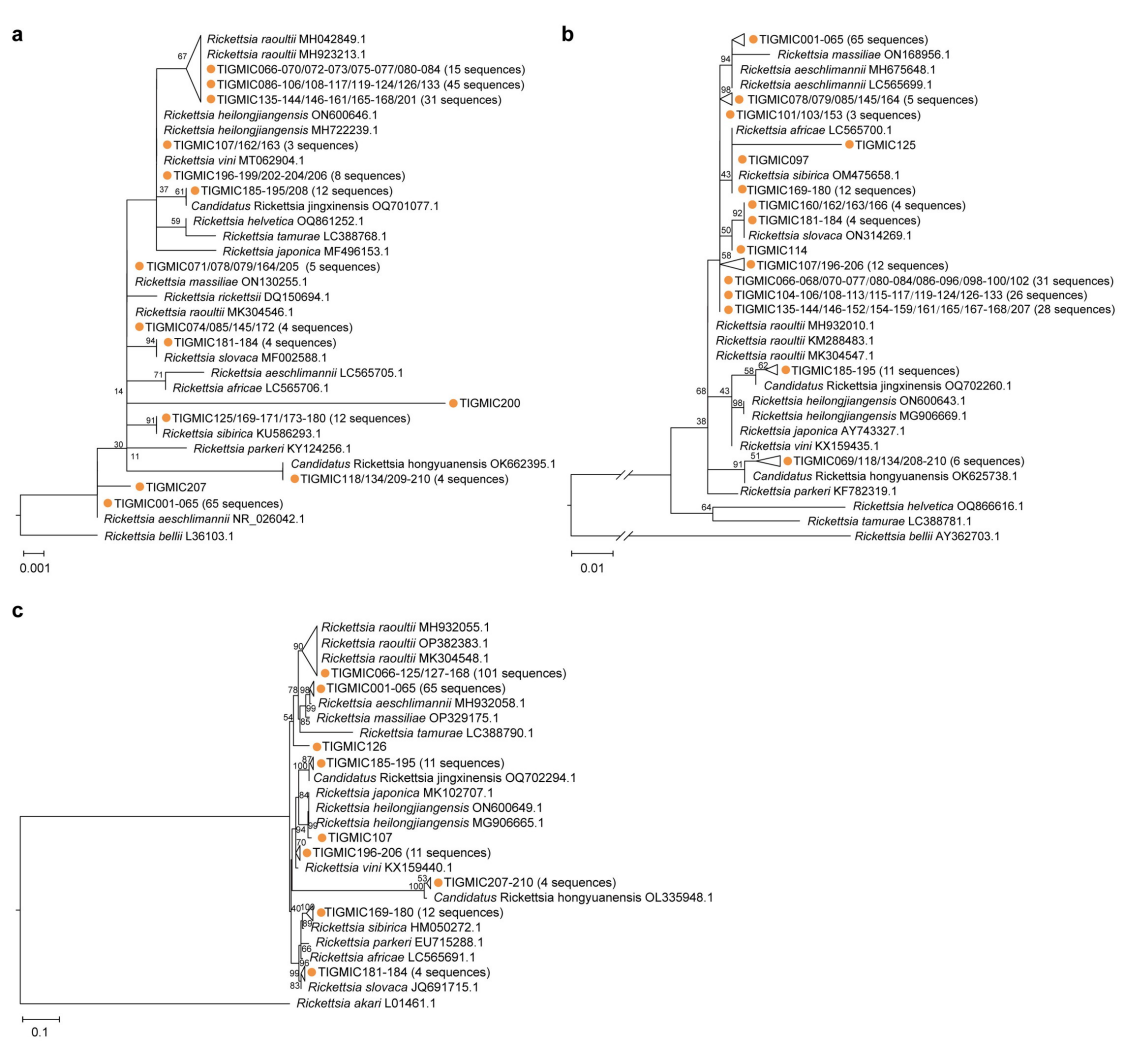
Phylogenetic trees of *Rickettsia* spp. based on the sequences of 3 different genes. The trees were constructed based on the nucleotide sequences of (a) *rrs* (760 bp), (b) *gltA* (381 bp) and (c) *ompA* (532 bp) using the maximum-likelihood method with the best substitution model found. All bootstrap support values from 1,000 replicates are shown at the interior branch nodes. The sequences obtained in this study are marked by orange circles.

We tested 425 adult ticks by nested or semi-nested PCR for the presence of SFGRs. In total, Rickettsial DNA were confirmed by Sanger sequencing in 210 (49.4%) of the 425 ticks. The gene sequences were subjected to BLAST analysis for a preliminary verification of their identity. BLAST results are shown in S4 Table. Phylogenetic trees based on the sequences of the *rrs*, *gltA* and *ompA* gene fragments with the ML method are shown in Fig 2. From the phylogenetic analysis based on three genes, most rickettsial sequences from this study clustered with *R*. *raoultii* and *R*. *aeschlimannii* isolates, some sequences of them were clustered together with *R*. *sibirica*, *R*. *slovaca*, *R*. *heilongjiangensis*, and *Ca*. R. hongyuanensis in branches, respectively. In addition, a few rickettsial sequences were most closely related to different SFGRs, even in separate clusters on these phylogenetic trees. For example, sample (TIGMIC125) clustered with *R*. *sibirica* (KU586293.1), *R*. *africae* (LC565700.1) and *R*. *raoultii* (MK304548.1) in the *rrs*, *gltA* and *ompA* phylogenetic trees, respectively (Fig 2).

### Phylogeny of rickettsiae based on the concatenation of the *rrs*, *ompA*, and *gltA* genes

**Fig 3:**
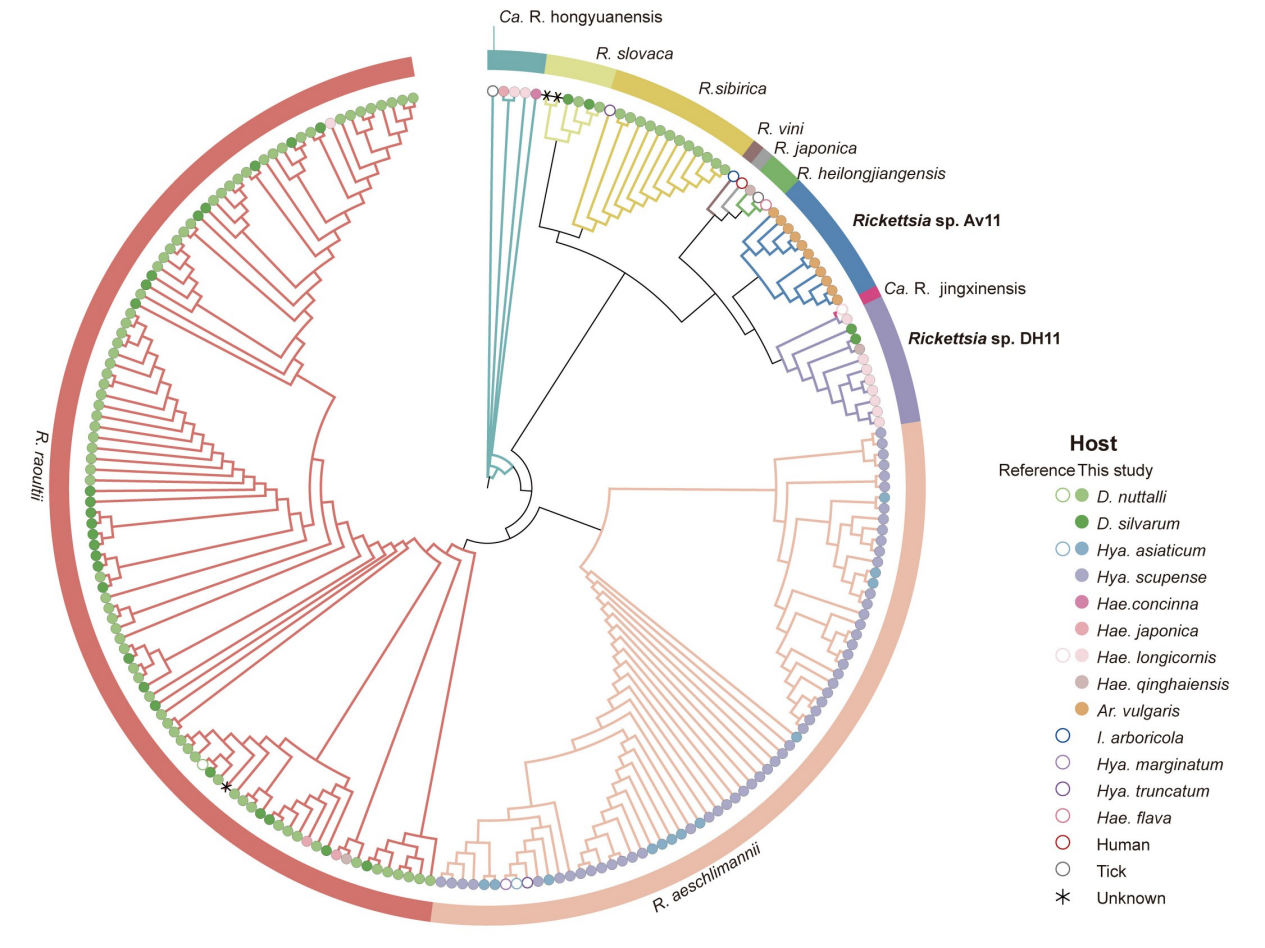
Phylogenetic tree of study samples with validated *Rickettsia* species. The partial nucleotide sequences of genes *rrs* (760 bp), *ompA* (532 bp), and *gltA* (381 bp) were concatenated and constructed via the maximum-likelihood method by using the best substitution model found. A bootstrap analysis of 1,000 replicates was applied to assess the reliability of the reconstructed phylogenies. Study sample *Rickettsia* sp. Av11 and *Rickettsia* sp. DH11 branched distinctly from other rickettsiae and were considered novel SFGR genotypes. The various coloured lines represent distinct pathogens. Circles indicate host sources; solid circles denote samples from this study, while the rest represent reference sequences.

For further characterization of the detected bacterial strains, the sequences of ticks positive for all three genes (*rrs*, *ompA* and *gltA*) were concatenated and aligned for rickettsial phylogenetic analysis. The concatenated sequences also included validated *Rickettsia* species available in GenBank (S2 Table). In total, 210 tick DNA samples tested positive for *Rickettsia* were available for analysis. The validated *Rickettsia* sequences based on concatenated tree which overlooked these clade credibility values was delineated into the eight species of SFGRs, including six known species: *R*. *raoultii*, *R*. *aeschlimannii*, *R*. *sibirica*, *R*. *slovaca*, *R*. *heilongjiangensis*, and *Ca*. Rickettsia hongyuanensis (Fig 3).

Among the ticks infected by *Rickettsia* species, the sequences of *R*. *raoultii* obtained in this study showed greater than 97.6% nucleotide (nt) identity with those of previously reported *R*. *raoultii* str. Khabarovsk isolated from *D*. *silvarum* in Russia and the *R*. *raoultii* isolate Datong-Dn-1 from *D*. *nuttalli* in China (Fig 3). *R*. *aeschlimannii* sequences had 99.7%–100% nt identity with multiple reference sequences (*R*. *aeschlimannii* isolates Baiyin-Ha-14, Baiyin-Hm-150, and RH15). The *R*. *sibirica* and *R*. *slovaca* strains detected in this study clustered together, comprising twelve and four positive samples, respectively. Sample sequences of *R*. *sibirica* shared more than 98.5% nt identity with *R*. *sibirica* str. RH05 isolated from *Hya*. *truncatum* in Senegal. Four sequences of *R*. *slovaca* identified in this study showed greater than 99.2% nt identity with *R*. *slovaca* strains 13-B and D-CWPP. *R*. *heilongjiangensis* was detected in only one sample (*Hae*. *qinghaiensis*), showing 99.3% nt identity with sequences from *R*. *heilongjiangensis* isolate XY-1 from *Hae*. *longicornis* in China.

Based on concatenated phylogenetic analysis, 22 samples from ticks with two novel SFGR genotypes with identical *rrs*, *gltA* and *ompA* gene sequences, which we designated *Rickettsia* sp. Av11 (TIGMIC196–206) and *Rickettsia* sp. DH11 (TIGMIC185–195). The sequences of 11 samples of *Rickettsia* sp. Av11 constituted an independent cluster on the concatenated tree, as a lone taxon between *R*. *vini* and *Ca*. R. jingxinensis, although showing exceeding 98.8% nt identity with the closest sequence from *R*. *vini* str. Boshoek1. *Rickettsia* sp. DH11. and *Ca*. R. jingxinensis comprise a separate cluster that appears most closely related to *R*. *vini*, *R*. *japonica* and *R*. *heilongjiangensis* (Fig 3). The *rrs*, *ompA*, and *gltA* sequences of SFGRs amplified from the tick samples were submitted to GenBank and assigned the accession numbers PP110549–PP110758 (*rrs*), PP117689–PP117898 (*ompA*), and PP150126–PP150335 (*gltA*) (S5 Table).

### Comparative analysis of phylogenies in novel rickettsiae

**Fig 4:**
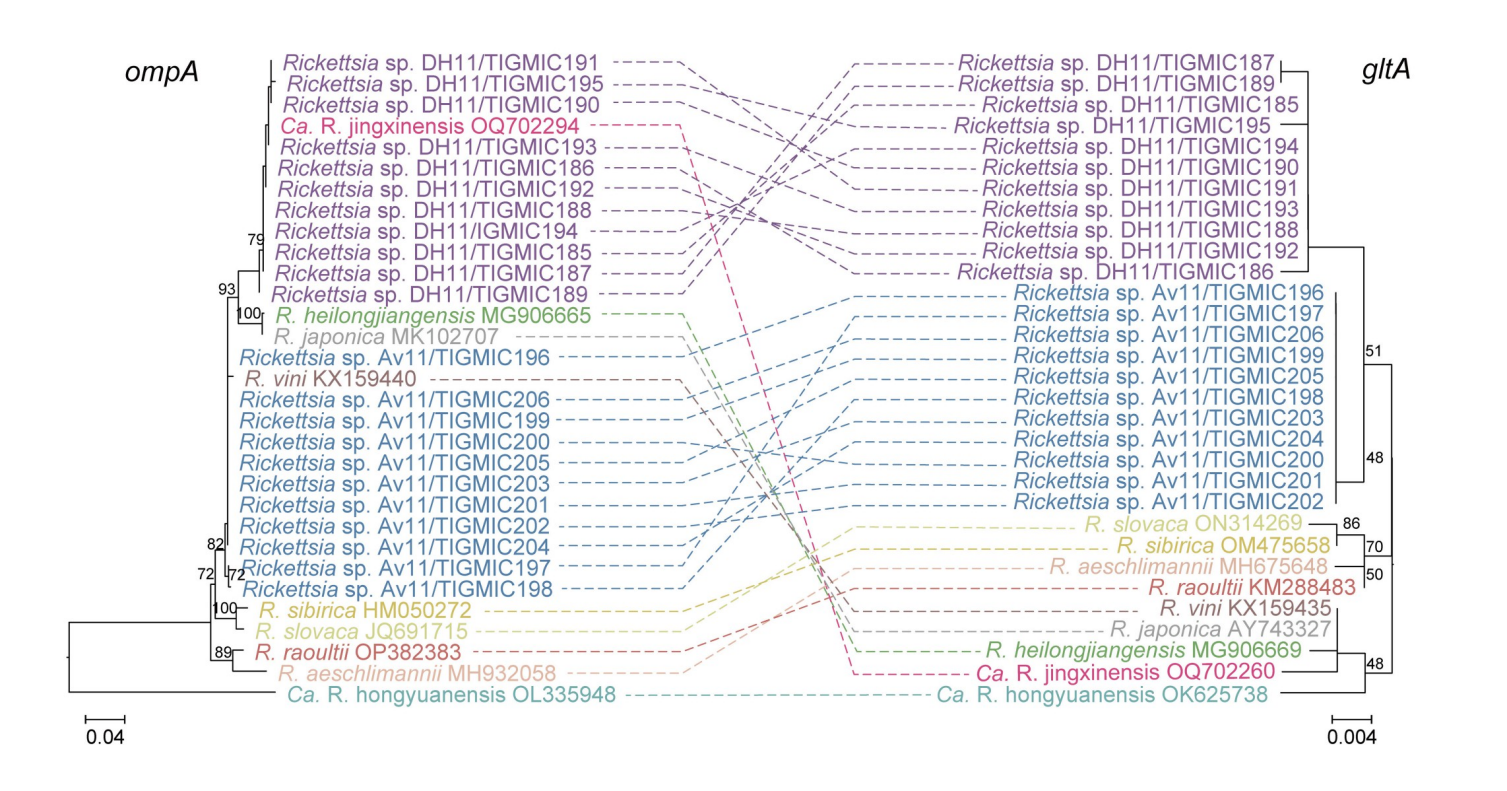
A comparative phylogenetic tree of SFGRs. Phylogenetic analysis based on nucleotide sequences of two protein-encoding genes (on the left: *ompA*, on the left: *gltA*). The numbers at the nodes are bootstrap proportions with 1000 replicates. The scale bar indicates the number of nucleotide substitutions per site.

In this study, we preliminary observed two possible new *Rickettsia* genotypes based on a phylogenetic tree constructed from concatenated sequences. To further describe the genetic characteristics of these new genotypes, single-gene segment phylogenetic trees were constructed based on the *rrs*, *gltA*, and *ompA* sequences of the new *Rickettsia* genotypes, and the results were comprehensively analyzed in conjunction with the concatenated sequence phylogenetic tree. Systematic analysis based on the *rrs* gene revealed a small genetic distance between branch sequences (S2 Fig), possibly due to the highly conservative nature of the *rrs* gene, which limits its effectiveness in species differentiation [47]. Therefore, we primarily referred to the phylogenetic trees based on the *ompA* and *gltA* genes and compared the two phylogenetic trees [48]. The sequences of *Rickettsia* sp. Av11 (TIGMIC196–206) in this study on the *ompA* phylogenetic tree were closest to *R*. *vini* (KX159440), showing 99.2%–99.4% identity, but formed an independent cluster on the *gltA* phylogenetic tree. A total of 11 sequences from *Rickettsia* sp. DH11 (TIGMIC185-195) were grouped together with *Ca*. R. jingxinensis (OQ702294) on the *ompA* phylogenetic tree, sharing 100% identity, whereas formed a separate branch on the *gltA* phylogenetic tree (Fig 4).

We amplified the *17kDa* gene again with these samples from the possible new *Rickettsia* genotypes were compared with those available in GenBank. For the SFG *Rickettsia* sp. Av11, although the *rrs* gene was 100% identical to *R*. *japonica* str. LA16/2015 (CP047359.1), its *17kDa*, *ompA* and *gltA* genes show 99.7%, 99.0%, 99.2% nucleotide similarity to *R*. *heilongjiangensis* isolate 2022-Tick251 (PP116500.1) from *Hae*. *flava*, *R*. *vini* str. Boshoek1 (MT062907.1) from *Ixodes arboricola* and Uncultured Rickettsia sp. isolate S6 (LC060714.1) from *D*. *reticulatus*, respectively. In the phylogenetic trees, its *rrs* and *ompA* genes were apparently divergent from other SFG *Rickettsia* species (Figs 3 and S2). Notably, its *gltA* gene is in a basal location in the phylogenetic tree but far from SFG *Rickettsia* species (Fig 3). According to the gene sequence-based criteria for taxonomic classification of new *Rickettsia* isolates, a *Candidatus* status could be assigned to Av11, so we named this species *Candidatus* Rickettsia vulgarisiii. For the *Rickettsia* sp. DH11, its *rrs*,*ompA*, *gltA* and *17kDa* genes all show above 99.5% nucleotide similarity to *Ca*. R. jingxinensis (MH500194.1, MN463682.1, OR801782.1 and MW114879). Given that phylogenetic analysis of both *rrs* and *ompA* gene sequences revealed that the *Rickettsia* sp. DH11 strains were clustered with *Ca*. R. jingxinensis, so this SFG species was identified as *Ca*. R. jingxinensis.

#### Detection of *Anaplasma* spp

**Fig 5:**
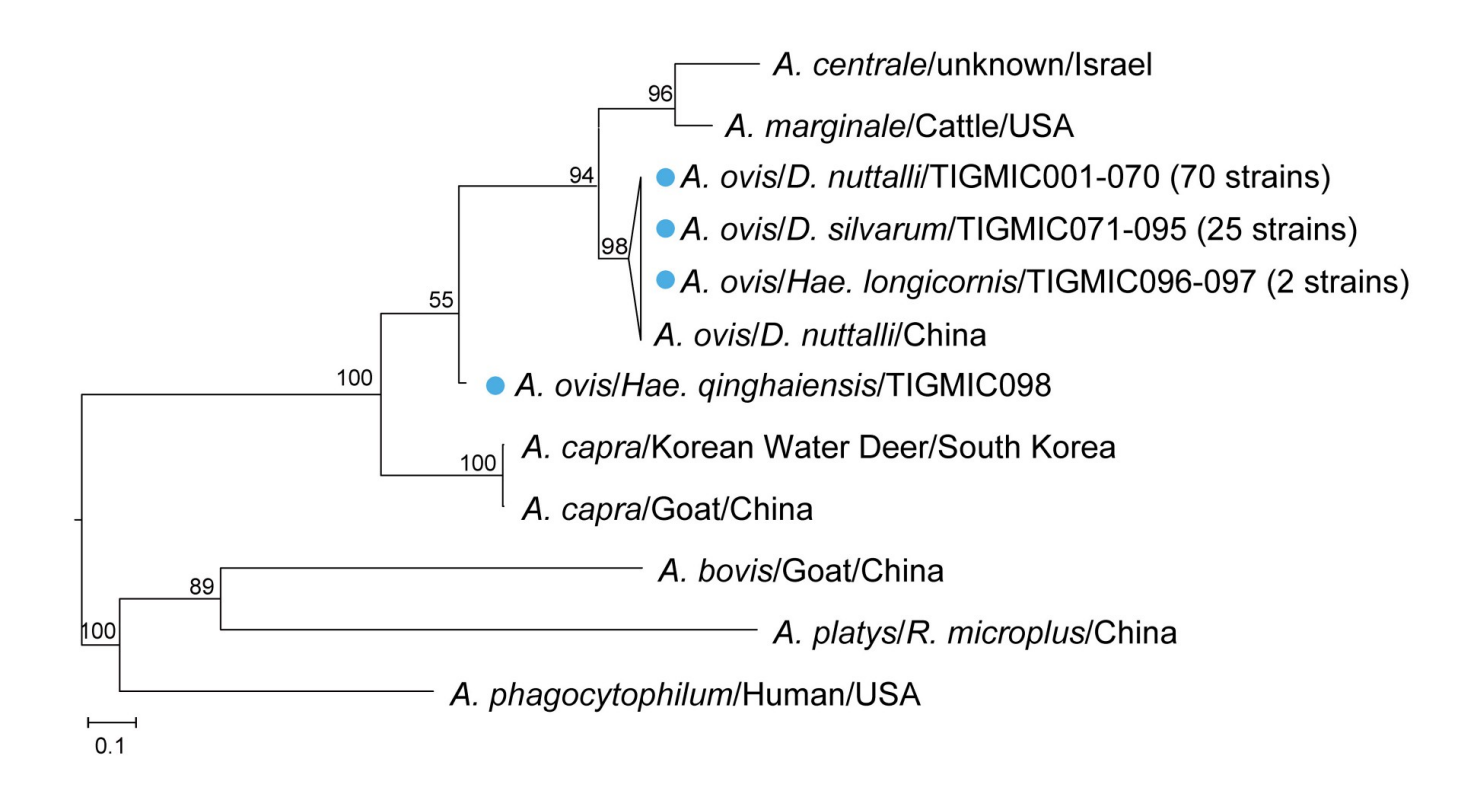
Phylogenetic tree of study samples with validated *Anaplasma* species. The tree was based on concatenated *rrs* (660 bp), *gltA* (793 bp), and *groEL* (1100 bp) nucleotide sequences. The tree was constructed by the maximum-likelihood method and we performed bootstrap analysis of 1,000 replicates to assess the reliability of the constructed phylogenies. The numbers at the nodes are bootstrap proportions with 1000 replicates. The scale bar indicates the number of nucleotide substitutions per site. The sequences obtained in this study are marked by blue circles.

To screen for *Anaplasma* infection in ticks, we also amplified the *rrs*, *groEL*, and *gltA* gene segments using nested PCR and detected 98 samples for all three positive genes. BLAST results are shown in S6 Table and phylogenetic trees based on single gene are shown in S3 Fig. As mentioned above, phylogenetic tree for *Anaplasma* species was also constructed by concatenated sequences from the three genes [49]. Among all positive sequences, 97 sample (TIGMIC001–097) showed 97.1%–100% identity to the *Anaplasma ovis* isolate TC249-5 detected in *D*. *nuttalli* from China, while the only remaining sample (TIGMIC098) fell between the two branches of *A*. *ovis* and *A*. *capra* (Fig 5), with 86.2% and 89.3% identity, respectively. Specific gene segments for *A*. *ovis* and *A*. *capra* were amplified again for this one sample, and only aligned with *A*. *ovis* (MH790273), showing 99.6% identity. This positive sample was identified as *A*. *ovis*. The nucleotide sequences of *Anaplasma rrs*, *groEL*, and *gltA* genes amplified from tick samples were submitted to GenBank with accession numbers PP106263–PP106360 (*rrs*), PP117399–PP117496 (*groEL*), and PP117094–PP117191 (*gltA*) (S5 Table).

### Prevalence of SFGRs and *Anaplasma* in Ticks

We screened 425 adult ticks from Ningxia region and *Rickettsia* and *Anaplasma* DNA were detected in 210 ticks and 98 ticks. The distribution of bacterial species and quantity is shown in S4 Fig. The most species and the highest positive number of SFGRs were in Guyuan city (65/210), while the highest positive rate was in Zhongwei city (68.8%, 53/77); the highest positive number and positive rate of *Anaplasma* species were in Guyuan city (50/98) and Wuzhong city (40.3%, 27/67), respectively (S4 Fig and S7 Table). We detected seven described species of *Rickettsia* and one novel candidate *Rickettsia* species (Figs 2–4). Sequencing of the *Anaplasma* amplicon determined the presence of one *Anaplsma* species in *D*. *nuttalli*, *D*. *silvarum*, *Hae*. *longicornis* and *Hae*. *qinghaiensis* (Fig 5). Eight species of *Rickettsia* were identified, while *R*. *raoultii* exhibited the highest infection rate (24.0%, 102/425), and *R*. *heilongjiangensis* had the lowest infection rate (0.2%, 1/425), detected solely in only one sample from *Hae*. *qinghaiensis* (Table 1). The prevalence of *R*. *raoultii*, the most abundant species, in *Dermacentor* ticks (52.7%, 98/186) was significantly higher than that in *Haemaphysalis* (3.2%, 4/124), *Hyalomma* (0/101), and *Argas* (0/14) ticks (χ^2 =^ 149.6, *df* = 3, *P* < 0.001). *R*. *aeschlimannii* was exclusively detected in *Hyalomma* ticks (64.4%, 65/101), *R*. *sibirica* and *R*. *slovaca* were solely identified in *Dermacentor* ticks, *Ca*. R. hongyuanensis identified in *Haemaphysalis* ticks, whereas *Ca*. R. jingxinensis was detected *in Dermacentor* and *Haemaphysalis* ticks. The infection rates of the newly discovered SFGR was relatively high in *Argas* ticks (78.6%, 11/14). Furthermore, 98 samples tested positive for *Anaplasma* species, showing an overall positivity rate of 23.1% (95% CI: 19.1–27.1), all of which identified as *A*. *ovis* (Table 2). *Anaplasma* infection was detected only in *Dermacentor* and *Haemaphysalis* ticks (51.1%, 95/186, 2.4%, 3/124), with no evidence in other tick species. The infection rate of *A*. *ovis* in *Dermacentor* ticks was significantly higher than that in *Haemaphysalis* ticks and other tick genera (χ^2^ = 146.5, *df* = 3, *P* < 0.001). We identified the greatest richness of bacteria strains in *D*. *nuttalli*.

**Table 1:** Prevalence of rickettsiae in ticks from Ningxia, China*.

Tick species	No. of tested	Positive rate (95% CI)								Total
		*R*. *raoultii*	*R*. *aeschlimannii*	*R*. *sibirica*	*R*. *slovaca*	*R*. *heilongjiangensis*	*Ca*. R. hongyuanensis	*Ca*. R. vulgarisii	*Ca*. R. jingxinensis	
*D*. *nuttalli*	121	60.3 (51.6–69.1)	0	9.9 (5.5–17.0)	1.7 (0.3–6.4)	0	0	0	0	71.9 (63.9–79.9)
*D*. *silvarum*	65	38.5 (26.6–50.3)	0	0	3.1 (0.5–11.7)	0	0	0	3.1 (0.5–11.7)	44.6 (32.5–56.7)
*Hya*. *asiaticum*	24	0	50.0 (30.0–70.0)	0	0	0	0	0	0	50.0 (30.0–70.0)
*Hya*. *scupense*	77	0	68.8 (58.5–79.2)	0	0	0	0	0	0	68.8 (58.5–79.2)
*Hae*. *concinna*	10	0	0	0	0	0	10.0 (0.5–45.9)	0	0	10.0 (0.5–45.9)
*Hae*. *japonica*	36	5.6 (1.0–20.0)	0	0	0	0	2.8 (0.2–16.2)	0	0	8.3 (2.2–23.6)
*Hae*. *longicornis*	42	2.4 (0.1–14.1)	0	0	0	0	4.8 (0.8–17.4)	0	19.1 (7.2–30.9)	26.2 (14.4–42.3)
*Hae*. *qinghaiensis*	36	2.8 (0.2–16.2)	0	0	0	2.8 (0.2–16.2)	0	0	2.8 (0.2–16.2)	8.3 (2.2–23.6)
*Ar*. *vulgaris*	14	0	0	0	0	0	0	78.6 (57.1–100)	0	78.6 (57.1–100.0)
Total	425	24.0 (19.9–28.1)	15.3 (11.9–18.7)	2.8 (1.5–5.0)	0.9 (0.3–2.6)	0.2 (0–1.5)	0.9 (0.3–2.6)	2.6 (1.4–4.7)	2.6 (1.1–4.1)	49.4 (44.7–54.2)

*Values are % (95% confidence interval) except where indicated.

**Table 2:** Prevalence of *Anaplasma ovis* in ticks from Ningxia, China.

Tick species	No. of tested	No. of positive	% (95% CI)
*D*. *nuttalli*	121	70	57.9 (49.1–66.6)
*D*. *silvarum*	65	25	38.5 (26.6–50.3)
*Hya*. *asiaticum*	24	0	0
*Hya*. *scupense*	77	0	0
*Hae*. *concinna*	10	0	0
*Hae*. *japonica*	36	0	0
*Hae*. *longicornis*	42	2	4.8 (0.8–17.4)
*Hae*. *qinghaiensis*	36	1	2.8 (0.2–16.2)
*Ar*. *vulgaris*	14	0	0
Total	425	98	23.1 (19.1–27.1)

Co-infection of *Rickettsia*-*Anaplasma* within individual ticks was observed in 14.6% (62/425) of the ticks tested. Co-infections of *R*. *raoultii* + *A*. *ovis* were 11.5% (49/425), *R*. *sibirica* +*A*. *ovis* were 2.4% (10/425), and *R*. *slovaca* + *A*. *ovis* were 0.7% (3/425). All co-infected samples were *Dermacentor* ticks, with co-infection rate of 38.0% (46/121) in *D*. *nuttalli* and 24.6% (16/65) in *D*. *silvarum* (Table 3).

**Table 3:** The number of positive co-infections in ticks from Ningxia.

Co–infections	No. (%, 95% CI)		Total
	*D*. *nuttalli*, n = 121	*D*. *silvarum*, n = 65	
*R*. *raoultii* +*A*. *ovis*	35 (28.9, 21.2–38.0)	14 (21.5, 12.7–33.8)	49 (26.3, 20.3–33.3)
*R*. *sibirica* +*A*. *ovis*	10 (8.26, 4.3–15.0)	0	10 (5.4, 2.8–10.0)
*R*. *slovaca* +*A*. *ovis*	1 (0.8, 0–5.2)	2 (3.1, 0.5–11.7)	3 (1.6, 0.4–5.0)
Total	46 (38.0, 29.5–47.3)	16 (24.6, 15.1–37.1)	62 (33.3, 26.7–40.7)

No. Number of positive samples, 95% CI 95% confidence interval

### Vector host preference of rickettsiae

**Fig 6:**
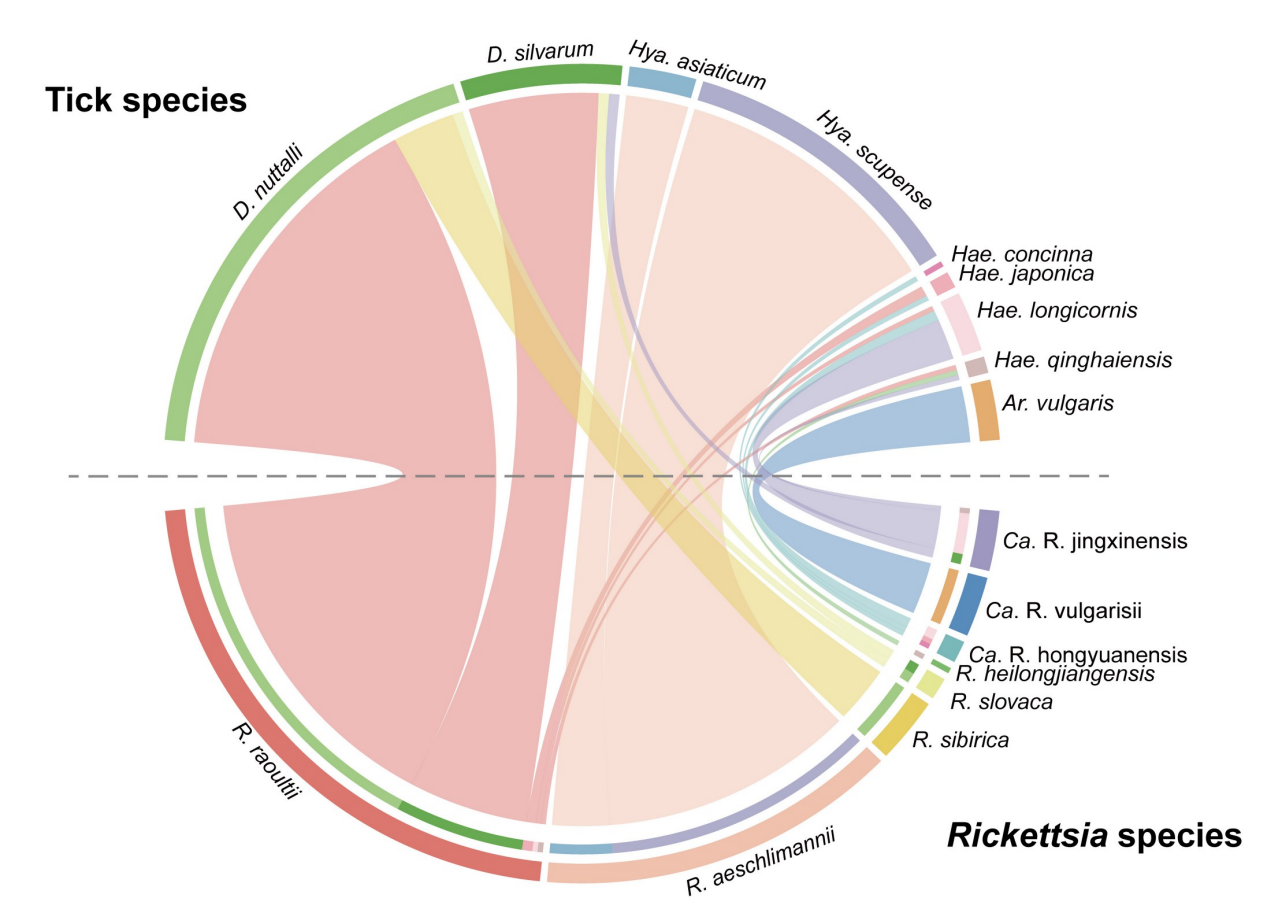
Each tick samples infected with *Rickettsia* species. The upper semicircle is the tick species, the lower semicircle is the *Rickettsia* species, and the color of the inner lines represent the tick species. The width of each chord corresponds to the count of *Rickettsia*-positive individuals in respective tick species.

The above analysis results revealed differences in the parasitic adaptability of various *Rickettsia* species to tick hosts. To further describe this phenomenon, a species correlation analysis was conducted on all the samples and *Rickettsia* species included in this study. As illustrated in the chord diagram (Fig 6), these *Rickettsia* species exhibited specificity to tick genera or species. *R*. *raoultii* demonstrated relatively high adaptability to various tick species, being detected in three *Haemaphysalis* species and two *Dermacentor* species, with a positive rate reaching 60.3% in *D*. *nuttalli*, followed by *D*. *silvarum* (38.5%). *R*. *aeschlimannii* was found in both *Hya*. *asiaticum* and *Hya*. *scupense*, with positive rates of 50.0% and 68.8% (Table 1), respectively, and was not detected in tick genera other than *Hyalomma* ticks. *R*. *sibirica* and *R*. *slovaca* were exclusively found in *Dermacentor* ticks, *Ca*. R. vulgarisii was exclusively detected in *Ar*. *vulgaris*, whereas *Ca*. R. hongyuanensis was solely found in three species of *Haemaphysalis* ticks. While *Ca*. R. jingxinensis displayed a lower positive rate than *R*. *raoulti*, it was also detected in different tick species. These findings suggest that the distinct host preferences and adaptation ranges exhibited by different *Rickettsia* species.

## Discussion

*Rickettsia* species exhibit broad pathogenicity, with some posing a lethal threat to humans [50], and ticks are critical vectors in the transmission cycle of these pathogens, affecting both human and animal health. Understanding the tick-host-pathogen interactions is essential for developing effective strategies to mitigate the impact of tick-borne diseases. Our results document the first detection in ticks of SFGRs and *Anaplasma* spp. collected from Ningxia, China. As a result, one *Anaplasma* species and eight SFGR species were identified in nine tick species. Sequence data provided evidence for the presence of *A*. *ovis*, *R*. *raoultii*, *R*. *aeschlimannii*, *R*. *sibirica*, *R*. *slovaca*, *R*. *heilongjiangensis*, *Ca*. R. hongyuanensis, *Ca*. R. jingxinensis, and one novel *Rickettsia* species, which we named “*Ca*. R. vulgarisii”.

SFGRs compose the important group in the genus *Rickettsia*. They are common tick-borne pathogens and have long been considered as the causative agents of various zoonotic diseases. In this study, a combination of single gene and concatenated genes (*rrs*, *ompA*, and *gltA*) were used to infer the evolutionary topology of *Rickettsia* using DNA samples obtained from ticks [51]. Eight *Rickettsia* species, especially a novel SFGR species named *Ca*. R. vulgarisii, were identified in ticks. Since its characterization in 2008 [52], *R*. *raoultii*, as a causative agent of human tick-borne lymphadenitis, has been reported in ticks from the Romania [24], India [53] and China [54]. The second SFGR identified in this study was *R*. *aeschlimannii*, which has been found in *Hya*. *marginatum* collected from southern Europe [55], and Africa [56]. *R*. *sibirica*, *R*. *slovaca* and *R*. *heilongjiangensis* are recognized as human pathogens that can cause mild rash associated with fever and eschars [57]. *Ca*. R. hongyuanensis and *Ca*. R. jingxinensis, belonging to SFG *Rickettsia*, were first identified in *Hae*. *longicornis* in southwest [58] and northeast China [59]. These findings indicate a human infection risk of important SFGR tick-borne diseases in the region.

Furthermore, we had detected an incompletely described *Rickettsia* in *Argas* ticks. This *Rickettsia* species had been detected with a highly positive rate in Zhongwei city, and had provisionally been named *Rickettsia* sp. Av11. The analysis of *rrs*, *ompA* and *gltA* genes and concatenated sequences confirmed the presence of this SFGR in Ningxia. As stated by de Sousa et al. [60], when they reported the detection of this bacterium, other gene sequences were required to establish its identity correctly according to the genetic guidelines published by Fournier et al. [61,62]. Genetic analyses indicate that its *rrs*, *ompA*, *gltA* and *17kDa* genes have the highest identities to different validated species and these stains in different trees were apparently divergent from other SFG *Rickettsia* species. Considering these criteria, we propose to give to this strain a *Candidatus* status and name it “*Ca*. R. vulgarisii”, with reference to the name *Ar*. *vulgaris* ticks in which this *Rickettsi*a species had been detected. *Argas* ticks can easily transmit and acquire bacteria to and from different hosts during its life cycle on account of the fact that they feed multiple times during a given developmental stage [63]. Wild birds, as the common hosts of *Argas* ticks, may be the non-negligible reason for introducing ticks and related pathogens into new environments due to their special migratory behaviors [64]. The role of *Ar*. *vulgaris* in the transmission of SFGRs should be further considered, and investigations on the human pathogenicity of these *Rickettsia* species are still needed. This discovery suggested that the potential threat of novel species to humans and animals still can not be excluded although its transmissibility and potential pathogenicity should be further studied.

*A*. *ovis* has been recognized as a tick-borne obligatory intraerythrocytic pathogen mainly infecting the ovine and caprine erythrocytes [65,66]. This pathogen can cause ovine anaplasmosis characterised by subclinical signs such as weakness, anorexia, weight loss and anaemia [67]. *A*. *ovis* was reported in all continents and has a widespread distribution in China [68], France [69] and Italy [70]. In this study, we observed remarkably high positivity rates of *A*. *ovis* in *D*. *nuttalli* (58.9%) and *D*. *silvarum* (45.7%). *Hae*. *longicornis* and *Hae*. *qinghaiensis* also tested positive for this *A*. *ovis* strain. Although the possibility cannot be ruled out that the *A*. *ovis* DNA may come from the blood meal of ticks, we suspect that *Dermacentor* ticks might play an important role in the transmission of *A*. *ovis* in domestic animals. The existence of *A*. *ovis* in Ningxia may indicate the risk of human infection and highlight the importance of surveillance in local populations though the pathogenicity of these strains to humans is still to be determined.

Evaluation of SFGRs and *Anaplasma* spp. prevalence at tick level was possible through phylogenetic analysis inference. The infection rates of SFGR and *Anaplasma* spp. in ticks from the Ningxia region were 49.4% and 23.1% in the present study and their positive rate among different tick species existed significance difference. The ticks were collected during the peak activity period of ticks, and *Dermacentor* ticks were the dominant tick genus, suggesting their important role in the distribution and transmission of rickettsiae in this region. The positive sequences of SFGRs and *A*. *ovis* in this study were mostly derived from ticks feeding on animal hosts (sheep and goat) rather than free ticks. These pathogens were found in regions with high biodiversity where migratory birds may inhabit. Combined with extensive livestock husbandry in Ningxia, they might spread to other hosts through these domestic animals. Previous studies have shown that mammals including sheeps and goats, particularly rodents and other small mammals, are often key reservoirs for tick-borne pathogens [2,21–23]. Meanwhile, studies have shown that a higher diversity of mammalian hosts can influence the transmission dynamics of these pathogens, reducing the transmission rates of pathogens like *A*. *phagocytophilum* [71]. This is due to the presence of non-susceptible species that can disrupt the cycle of transmission among more susceptible hosts. The ecological and economic factors in the Ningxia region have led to a rich array of animal resources. Therefore, according to the One health concept, it is not only human infections that are of concern, but infections in wild animals and livestock are also major health issues that deserve attention [72].

Our results also showed that co-infection of *A*. *ovis* and SFGR (*R*. *raoultii*, *R*. *sibirica* and *R*. *slovaca*) existed in *Dermacentor* ticks, and suggested potential interactions between these pathogens within tick vectors. The results obtained in the current study coincide with those previously described in Thailand, which reported co-infection with *Rickettsia* spp. and *Anaplasma* spp. in *Dermacentor* ticks [73]. It is reported that persistent infection of *A*. *ovis* can be accompanied by cyclical fluctuations in rickettsial levels, which may obviously alter the vector infection rate and thus alter transmission [74]. Previous research also indicated that co-infection often leads to a broader spectrum of clinical symptoms, prolongs disease duration, and exacerbates disease severity [75]. For instance, co-infection with both *Borrelia burgdorferi* and *A*. *phagocytophilum*, as opposed to single infections of either one, leads to more deep impairment of endothelial barrier function [76]. The higher co-infection rate in *Dermacentor* ticks, particularly *D*. *nuttalli*, suggests potential synergistic or competitive dynamics between these two genera of pathogens. The interactions between co-infections of pathogens within ticks and the impact of tick-borne transmission on human health still require further study [77].

Due to its intracellular lifestyle, rickettsiae are highly dependent on their primary tick vectors and tend to be selective for the tick species they infect. In this study, most bacteria were detected in *Dermacentor* ticks. Meanwhile, some bacteria were also detected in *Haemaphysalis* ticks, *Hyalomma* and *Argas* ticks. Further analysis of host preferences among different *Rickettsia* species highlighted differences in adaptability to various tick species. In this study, *R*. *raoultii* displayed relatively high adaptability across five tick species, with infections in *Dermacentor* ticks accounting for as much as 96.1% (98/102) of the tick population. In contrast, other *Rickettsia* species exhibited more specific host preferences. *R*. *aeschlimannii* showed a preference for two species of *Hyalomma* ticks. Among the newly discovered *Rickettsia*, *Ca*. R. vulgarisii was detected only in *Ar*. *vulgaris*, further emphasizing the genus-specific adaptation of *Rickettsia* species. Additionally, the detection of *Anaplasma* species in ticks also indicated that they have varying adaptability to different tick species. Beyond the *Rickettsia* and *Anaplasma* species studied herein, ticks generally engage in complex ecological interactions with their microbiota [78], where the presence of specific pathogens contributes to the stability of tick microbial communities and influences their population dynamics and metabolic functions [79,80]. The adaptability of *Rickettsia* species to specific hosts holds significant value in maintaining the structure and function of pathogen-vector microbial communities [81], suggesting that research on the mechanisms of co-evolution between ticks and the microbiota will enhance efforts to control the risk of *Rickettsia* disease transmission.

These findings presented here are of epidemiological importance. We have characterized a novel SFGR named “*Ca*. R. vulgarisii”, which may enhance our understanding of our knowledge on the diversity of SFGRs. Although the human pathogenicity for this novel bacterium from a limited sources of *Argas* ticks is still determined, more attention should be paid to the risk of human infection and the possible circulation of these pathogens in local population. Thus, further studies are needed to explore its implications for human and/or animal diseases in Ningxia by extending ecological surveys with an increased number of tick species and tick specimens. The host range, distribution, and pathogenicity of “*Ca*. R. vulgarisii” also merit further investigations.

## Conclusions

In conclusion, the present study provided a molecular detection on *Rickettsia* spp. and *Anaplasma* spp. for the first time in ticks from Ningxia, northwestern China. One *Anaplasma* species (*A*. *ovis*) and egiht SFGR (*R*. *raoultii*, *R*. *aeschlimannii*, *R*. *sibirica*, *R*. *slovaca*, *R*. *heilongjiangensis*, *Ca*. R. hongyuanensis, *Ca*. R. jingxinensis and *Ca*. R. vulgarisii) including a novel species were detected and characterized. Our findings reveal a presence of diverse SFGRs including unidentified agents within tick species in Ningxia. Further investigations should be focused on expanding the sampling range, required to examine the effects of ecological and seasonal factors on ticks and pathogens, and ascertain the pathogenicity of newly emerged SFGRs in humans. Moreover, variations in host adaptability among different *Rickettsia* species highlight the complexity of the transmission and dissemination of tick-borne diseases. Therefore, there is a compelling need for intensified monitoring of tick and tick-borne pathogens in subsequent research attempt.

## Supporting information

S1 Table Nucleotide sequence of primers used for detecting ticks from Ningxia, China. (DOCX)

S2 Table GenBank accession numbers for validated strains used for concatenated sequence. (DOCX)

S3 Table Tick samples tested in this study and their location. (DOCX)

S4 Table Identity of positive sequence of detected *Rickettsia* spp. with BLAST analysis. (DOCX)

S5 Table Sequences of *Rickettsia* and *Anaplasma* from tick samples deposited in GenBank. (DOCX)

S6 Table Identity of positive sequence of *Anaplasma* spp. with BLAST analysis. (DOCX)

S7 Table Prevalence of rickettsiae and *Anaplasma ovis* in ticks from different cities of Ningxia. (DOCX)

S1 Fig Distribution of *Dermacentor* samples as the dominant tick species collected in Ningxia, China. Different colour and size of circles represent the species and number of ticks collected from Ningxia. The map was constructed using ArcGIS v10.8.2 software. The basemap shapefiles were downloaded from the Chinese Resource and Environmental Science Data Platform (http://www.resdc.cn/, DOI:10.12078/2023010102).
(TIF)

S2 Fig Phylogenetic analysis of unidentified *Rickettsia* (22 samples) based on the 760bp nucleotide sequence of *rrs* gene. Numbers at the nodes are bootstrap proportions with 1000 replicates. The scale bar indicates the number of nucleotide substitutions per site.
(TIF)

S3 Fig Phylogenetic trees of Anaplasma species based on the sequences of 3 different genes. The trees were constructed based on the nucleotide sequences of (a) *rrs* (660 bp), (b) *gltA* (793 bp) and (c) *groEL* (1100 bp) using the maximum-likelihood method with the best substitution model found. All bootstrap support values from 1,000 replicates are shown at the interior branch nodes. The sequences obtained in this study are marked by blue circles.
(TIF)

S4 Fig Distribution of *Rickettsia* and *Anaplasma* samples detected in Ningxia, China. Different colour and size of circles (and cross symbols) represent the species and number of *Rickettsia* and *Anaplasma* detected from ticks. The map was constructed using ArcGIS v10.8.2 software. The basemap shapefiles were downloaded from the Chinese Resource and Environmental Science Data Platform (http://www.resdc.cn/, DOI:10.12078/2023010102).
(TIF)
