# Supplementary material for: The first direct detection of spotted fever group Rickettsia spp. diversity in ticks from Ningxia, northwestern China
Source: PLoS Negl Trop Dis. 2025 Jan 2;19(1):e0012729. doi: 10.1371/journal.pntd.0012729 (PMC11695002; doi:10.1371/journal.pntd.0012729)
Supplement: S1 Table — (DOCX) [file pntd.0012729.s001.docx]

**S1 Table.** Nucleotide sequence of primers used for detecting ticks from Ningxia, China.

| Species | Target | Primer name | Primer sequence (5'–3') | Annealing temperature (℃) | Amplicon  size (bp) | Note |
| --- | --- | --- | --- | --- | --- | --- |
| *Rickettsia* spp. | *rrs* | 16s20 | GACGGGTGAGTAACACGTGGG | 60 | 1140 | first–PCR |
|  |  | 16s1160 | GTCTTTTAGGGATTTGCTCCAC |  |  |  |
|  |  | 16s120 | GATGGATGAGCCCGCGTCAG | 60 | 760 | nested–PCR |
|  |  | 16s880 | GCATCTCTGCGATCCGCGAC |  |  |  |
|  | *gltA* | CS2d | ATGACCAATGAAAATAATAAT | 50 | 1100 | first–PCR |
|  |  | CSEndr | CTTATACTCTCTATGTACA |  |  |  |
|  |  | RpCS877f | GGGGACCTGCTCACGGCGG | 52 | 381 | nested–PCR |
|  |  | RpCS1258r | ATTGCAAAAAGTACAGTGAACA |  |  |  |
|  | *ompA* | 70F | ATGACGAATATTTCTCCAAAA | 50 | 631 | first–PCR |
|  |  | 701R | GTTCCGTTAATGGCAGCATCT |  |  |  |
|  |  | 70F | ATGACGAATATTTCTCCAAAA | 50 | 532 | semi nested–PCR |
|  |  | 602R | AGTGCAGCATTCGCTCCCCCT |  |  |  |
|  | *17kDa* | 17k3 | GCTTTACAAAATTCTAAAAACCATATA | 52 | 547 | first–PCR |
|  |  | 17k5 | TGTCTATCAATTCACAACTTGCC |  |  |  |
|  |  | Tara17KD13s1 | ATTGTCCGTCAGGTTGGC | 52 | 395 | nested–PCR |
|  |  | Tara17KD408r1 | CGGGCGGTATGAATAAGC |  |  |  |
| *Anaplasma* spp. | *rrs* | out–1 | TTGAGAGTTTGATCCTGGCTCAGAACG | 50 | 1500 | first–PCR |
|  |  | 3–17 | TAAGGTGGTAATCCAGC |  |  |  |
|  |  | out–1 | TTGAGAGTTTGATCCTGGCTCAGAACG | 50 | 660 | semi nested–PCR |
|  |  | out–2 | CACCTCTACACTAGGAATTCCGCTATC |  |  |  |
|  | *gltA* | Outer–f | GCGATTTTAGAGTGYGGAGATTG | 53 | 1077 | first–PCR |
|  |  | Outer–r | TACAATACCGGAGTAAAAGTCAA |  |  |  |
|  |  | Inner–f | GGGTTCMTGTCYACTGCTGCGTG | 53 | 793 | nested–PCR |
|  |  | Inner–r | TTGGATCGTARTTCTTGTAGACC |  |  |  |
|  | *groEL* | ovisge37f | AAATCTATAAGGGAGGTAGTGC | 50 | 1438 | first–PCR |
|  |  | ovisg1474r | CGTTAGCGTAGTTCATGGTG |  |  |  |
|  |  | ovisge37f | AAATCTATAAGGGAGGTAGTGC | 50 | 1100 | nested–PCR |
|  |  | ovisg1048r | GGCTAGTCCTGCTGGTAAT |  |  |  |
| *Anaplasma ovis* | *msp4* | ovismsp4f | GGGAGCTCCTATGAATTACAGAGAATTGTTTAC | 60 | 1000 | PCR |
|  |  | ovismsp4r | CCGGATCCTTAGCTGAACAGGAATCTTGC |  |  |  |
| *Anaplasma capra* | *groEL* | Ac–gF | GCGAGGCGTTAGACAAGTCCATT | 56 | 1264 | PCR |
|  |  | Ac–gR | TCCAGAGATGCAAGCGTGTATAG |  |  |  |
