# Supplementary material for: The first direct detection of spotted fever group Rickettsia spp. diversity in ticks from Ningxia, northwestern China
Source: PLoS Negl Trop Dis. 2025 Jan 2;19(1):e0012729. doi: 10.1371/journal.pntd.0012729 (PMC11695002; doi:10.1371/journal.pntd.0012729)
Supplement: S2 Table — (DOCX) [file pntd.0012729.s002.docx]

**S2 Table.** GenBank accession numbers for validated strains used for concatenated sequence

| Species | Strain | Genome (Accession no.) | GenBank accession no. | | | |
| --- | --- | --- | --- | --- | --- | --- |
|  |  |  | *rrs* | *gltA* | *ompA* | *groEL* |
| *R. raoultii* | Khabarovsk | CP010969 | † | † | † | NA |
| *R. raoultii* | Datong–Dn–1 | ‡ | MH923213.1 | MH932010.1 | MH932055.1 | NA |
| *R. aeschlimannii* | Baiyin–Ha–14 | ‡ | MH923216.1 | MH932013.1 | MH932058.1 | NA |
| *R. aeschlimannii* | Baiyin–Hm–150 | ‡ | MH923217.1 | MH932014.1 | MH932059.1 | NA |
| *R. aeschlimannii* | RH15 | ‡ | HM050274.1 | HM050283.1 | HM050277.1 | NA |
| *R. sibirica* | RH05 | ‡ | HM050271.1 | HM050296.1 | HM050272.1 | NA |
| *R. slovaca* | 13–B | CP002428 | † | † | † | NA |
| *R. slovaca* | D–CWPP | CP003375 | † | † | † | NA |
| *R. heilongjiangensis* | Huaian–HFL | ‡ | ON600646.1 | ON600643.1 | ON600649.1 | NA |
| *R. heilongjiangensis* | XY–1 | ‡ | MZ646340.1 | MZ646342.1 | MZ646345.1 | NA |
| *R. japonica* | MZ08014 | AP017577.1 | † | † | † | NA |
| *R. vini* | Boshoek1 | ‡ | MT062904.1 | MT062909.1 | MT062907.1 | NA |
| *Ca.* R. hongyuanensis | tick61 | ‡ | OK662395.1 | OK625738.1 | OL335948.1 | NA |
| *Ca.* R. jingxinensis | HBSJZPS105 | ‡ | OQ701077.1 | OQ702260.1 | OQ702294.1 | NA |
| *A. bovis* | Dongda–goat–210 | ‡ | MH255932.1 | MH594292.1 | NA | MH255908.1 |
| *A. capra* | KWD–18 | ‡ | LC432109.1 | LC432179.1 | NA | LC432144.1 |
| *A. capra* | BIME1 SDAC | GCA_025628785.1 | † | † | NA | † |
| *A. centrale* | Israel | NC_013532.1 | † | † | NA | † |
| *A. marginale* | Florida | NC_012026.1 | † | † | NA | † |
| *A. ovis* | TC249–5 | ‡ | KJ410245.1 | KJ410284.1 | NA | KJ410298.1 |
| *A. phagocytophilum* | HZ | NC_007797.1 | † | † | NA | † |
| *A. platys* | JZT2 | ‡ | OQ132527.1 | OQ185241.1 | NA | OQ185207.1 |

† = Complete genome available in GenBank;

‡ = Individual genes sequences available in GenBank;

NA = Gene sequences not available in GenBank.
