# Supplementary material for: The first direct detection of spotted fever group Rickettsia spp. diversity in ticks from Ningxia, northwestern China
Source: PLoS Negl Trop Dis. 2025 Jan 2;19(1):e0012729. doi: 10.1371/journal.pntd.0012729 (PMC11695002; doi:10.1371/journal.pntd.0012729)
Supplement: S3 Table — (DOCX) [file pntd.0012729.s003.docx]

**S3 Table.** Tick samples collected in this study and their location.

| City | Source | Sex | Tick species | | | | | | | | | Total |
| --- | --- | --- | --- | --- | --- | --- | --- | --- | --- | --- | --- | --- |
|  |  |  | *D. nuttalli* | *D. silvarum* | *Hya. asiaticum* | *Hya. scupense* | *Hae. concinna* | *Hae. japonica* | *Hae. longicornis* | *Hae. qinghaiensis* | *Ar. vulgaris* |  |
| Guyuan | sheep/goat/vegetation | 146♀, 70♂ | 27 | 65 | 0 | 0 | 10 | 36 | 42 | 36 | 0 | 216 |
| Shizuishan | goat | 51♀, 26♂ | 0 | 0 | 0 | 77 | 0 | 0 | 0 | 0 | 0 | 77 |
| Wuzhong | sheep | 49♀, 18♂ | 67 | 0 | 0 | 0 | 0 | 0 | 0 | 0 | 0 | 67 |
| Yinchuan | NA | NA | NA | NA | NA | NA | NA | NA | NA | NA | NA | NA |
| Zhongwei | sheep/goat/vegetation | 29♀, 36♂ | 27 | 0 | 24 | 0 | 0 | 0 | 0 | 0 | 14 | 65 |
| Total | sheep/goat/vegetation | 275♀, 150♂ | 121 | 65 | 24 | 77 | 10 | 36 | 42 | 36 | 14 | 425 |

NA = Tick not collected.
