# Supplementary material for: The first direct detection of spotted fever group Rickettsia spp. diversity in ticks from Ningxia, northwestern China
Source: PLoS Negl Trop Dis. 2025 Jan 2;19(1):e0012729. doi: 10.1371/journal.pntd.0012729 (PMC11695002; doi:10.1371/journal.pntd.0012729)
Supplement: S4 Table — (DOCX) [file pntd.0012729.s004.docx]

**S4 Table**. **Identity of positive sequence detected *Rickettsia* spp**. **with BLAST analysis**.

| City | Sample number | Animal source | Tick species | Sequence homology results, % | | |
| --- | --- | --- | --- | --- | --- | --- |
|  |  |  |  | *rrs* | *ompA* | *gltA* |
| Guyuan | TIGMIC066 | NA | *D*. *silvarum* | *R*. *raoultii* (MN446747.2), 100% | *R*. *raoultii* (MN450413.2), 100% | Uncultured *Rickettsia* sp. (MN431836.1), 100% |
| Guyuan | TIGMIC067 | NA | *D*. *silvarum* | *R*. *raoultii* (MN446747.2), 100% | *R*. *raoultii* (MN450413.2), 100% | Uncultured *Rickettsia* sp. (LC060714.1), 99.7% |
| Guyuan | TIGMIC068 | NA | *D*. *silvarum* | *R*. *raoultii* (MN446747.2), 99.9% | *R*. *raoultii* (MN450413.2), 100% | Uncultured *Rickettsia* sp. (LC060714.1), 99.2% |
| Guyuan | TIGMIC069 | NA | *Hae*. *longicornis* | *R*. *raoultii* (MN446747.2), 100% | *R*. *raoultii* (MN450412.2), 100% | Uncultured *Rickettsia* sp. (OR971697.1), 99% |
| Guyuan | TIGMIC089 | sheep | *D*. *nuttalli* | *R*. *raoultii* (MN446747.2), 100% | *R*. *raoultii* (MK304548.1), 99.8% | Uncultured *Rickettsia* sp. (LC060714.1), 99.7% |
| Guyuan | TIGMIC107 | sheep | *Hae*. *qinghaiensis* | *R*. *japoniCa* (CP047359.1), 100% | *R*. *japoniCa* (MK102719.1), 99.2% | Uncultured *Rickettsia* sp. (KY659474.1), 99.5% |
| Guyuan | TIGMIC108 | sheep | *D*. *silvarum* | *R*. *raoultii* (MN446747.2), 99.9% | *R*. *raoultii* (MK304548.1), 99.1% | Uncultured *Rickettsia* sp. (MN431836.1), 100% |
| Guyuan | TIGMIC109 | sheep | *D*. *silvarum* | *R*. *raoultii* (MN446747.2), 100% | *R*. *raoultii* (MK304548.1), 99.5% | Uncultured *Rickettsia* sp. (MN431836.1), 99.7% |
| Guyuan | TIGMIC110 | sheep | *D*. *silvarum* | *R*. *raoultii* (MN446747.2), 99.9% | *R*. *raoultii* (MK304548.1), 98.6% | Uncultured *Rickettsia* sp. (MN431836.1), 100% |
| Guyuan | TIGMIC111 | sheep | *D*. *silvarum* | *R*. *raoultii* (MN446747.2), 100% | *R*. *raoultii* (MK304548.1), 99.5% | Uncultured *Rickettsia* sp. (MN431836.1), 100% |
| Guyuan | TIGMIC112 | sheep | *D*. *silvarum* | *R*. *raoultii* (MN446747.2), 100% | *R*. *raoultii* (MK304548.1), 99.3% | Uncultured *Rickettsia* sp. (MN431836.1), 100% |
| Guyuan | TIGMIC113 | sheep | *D*. *silvarum* | *R*. *raoultii* (MN446747.2), 100% | *R*. *raoultii* (MK304548.1), 99.8% | Uncultured *Rickettsia* sp. (MN431836.1), 99.7% |
| Guyuan | TIGMIC114 | NA | *D*. *nuttalli* | *R*. *raoultii* (MN446747.2), 99.9% | *R*. *raoultii* (MK304548.1), 99.8% | Uncultured *Rickettsia* sp. (MN431836.1), 99.2% |
| Guyuan | TIGMIC115 | NA | *D*. *nuttalli* | *R*. *raoultii* (MN446747.2), 100% | *R*. *raoultii* (MN450413.2), 99.8% | Uncultured *Rickettsia* sp. (MN431836.1), 99.7% |
| Guyuan | TIGMIC116 | NA | *D*. *nuttalli* | *R*. *raoultii* (MN446747.2), 100% | *R*. *raoultii* (MK304548.1), 99.4% | Uncultured *Rickettsia* sp. (MN431836.1), 99.7% |
| Guyuan | TIGMIC117 | NA | *D*. *nuttalli* | *R*. *raoultii* (MN446747.2), 100% | *R*. *raoultii* (MK304548.1), 99% | Uncultured *Rickettsia* sp. (MN431834.1), 100% |
| Guyuan | TIGMIC118 | sheep | *Hae*. *qinghaiensis* | *Ca*. R. hongyuanensis (OK662395.1), 100% | *R*. *raoultii* (MK304548.1), 99.1% | Uncultured *Rickettsia* sp. (OR971697.1), 99.5% |
| Guyuan | TIGMIC133 | NA | *Hae*. *japonica* | *R*. *raoultii* (MT509815.1), 100% | *R*. *raoultii* (MN450413.2), 99.8% | Uncultured *Rickettsia* sp. (LC060714.1), 100% |
| Guyuan | TIGMIC134 | sheep | *Hae*. *japonica* | *Ca*. R. hongyuanensis (OK662395.1), 100% | *R*. *raoultii* (KX446992.1), 99.4% | *Ca*. R. principis (OR805124.1), 100% |
| Guyuan | TIGMIC135 | sheep | *D*. *silvarum* | *R*. *raoultii* (MN446747.2), 99.9% | *R*. *raoultii* (MN450413.2), 100% | Uncultured *Rickettsia* sp. (MN431834.1), 100% |
| Guyuan | TIGMIC136 | sheep | *D*. *silvarum* | *R*. *raoultii* (MN446747.2), 99.9% | *R*. *raoultii* (MN450413.2), 100% | Uncultured *Rickettsia* sp. (MN431836.1), 99.2% |
| Guyuan | TIGMIC137 | sheep | *D*. *silvarum* | *R*. *raoultii* (MN446747.2), 100% | *R*. *raoultii* (MN450413.2), 100% | Uncultured *Rickettsia* sp. (MN431836.1), 99.7% |
| Guyuan | TIGMIC140 | sheep | *D*. *nuttalli* | *R*. *raoultii* (MN446747.2), 99.9% | *R*. *raoultii* (MN450413.2), 100% | Uncultured *Rickettsia* sp. (MN431836.1), 100% |
| Guyuan | TIGMIC141 | sheep | *D*. *nuttalli* | *R*. *raoultii* (MN446747.2), 100% | *R*. *raoultii* (MN450413.2), 100% | Uncultured *Rickettsia* sp. (MN431836.1), 100% |
| Guyuan | TIGMIC142 | sheep | *D*. *nuttalli* | *R*. *raoultii* (MN446747.2), 99.7% | *R*. *raoultii* (MN450413.2), 100% | Uncultured *Rickettsia* sp. (LC060714.1), 100% |
| Guyuan | TIGMIC143 | sheep | *D*. *silvarum* | *R*. *raoultii* (MN446747.2), 100% | *R*. *raoultii* (MN450413.2), 100% | Uncultured *Rickettsia* sp. (LC060714.1), 99.7% |
| Guyuan | TIGMIC157 | NA | *D*. *silvarum* | *R*. *raoultii* (MN446747.2), 100% | *R*. *raoultii* (MN450413.2), 100% | Uncultured *Rickettsia* sp. (MN431836.1), 99.2% |
| Guyuan | TIGMIC158 | sheep | *D*. *silvarum* | *R*. *raoultii* (MN446747.2), 99.6% | *R*. *raoultii* (MN450413.2), 100% | Uncultured *Rickettsia* sp. (MN431836.1), 99.5% |
| Guyuan | TIGMIC159 | sheep | *D*. *silvarum* | *R*. *raoultii* (MN446747.2), 100% | *R*. *raoultii* (MN450413.2), 100% | Uncultured *Rickettsia* sp. (MN431836.1), 99.7% |
| Guyuan | TIGMIC160 | sheep | *D*. *silvarum* | *R*. *raoultii* (MN446747.2), 100% | *R*. *raoultii* (MN450413.2), 100% | Uncultured *Rickettsia* sp. (MG598409.1), 99.5% |
| Guyuan | TIGMIC161 | sheep | *D*. *silvarum* | *R*. *raoultii* (MN446747.2), 100% | *R*. *raoultii* (MN450413.2), 100% | Uncultured *Rickettsia* sp. (LC060714.1), 99.7% |
| Guyuan | TIGMIC162 | sheep | *D*. *silvarum* | *R*. *japoniCa* (CP047359.1), 100% | *R*. *raoultii* (KX506736.1), 97.3% | Uncultured *Rickettsia* sp. (MG598409.1), 99% |
| Guyuan | TIGMIC163 | sheep | *D*. *silvarum* | *R*. *japoniCa* (CP047359.1), 100% | *R*. *raoultii* (MN450413.2), 100% | Uncultured *Rickettsia* sp. (MG598409.1), 99.2% |
| Guyuan | TIGMIC164 | sheep | *D*. *silvarum* | *R*. *raoultii* (ON191634.1), 99.9% | Uncultured *Rickettsia* sp. (MG598411.1), 99.6% | Uncultured *Rickettsia* sp. (MN431836.1), 99.2% |
| Guyuan | TIGMIC165 | sheep | *D*. *silvarum* | *R*. *raoultii* (MN446747.2), 100% | *R*. *raoultii* (MN450413.2), 100% | Uncultured *Rickettsia* sp. (MN431836.1), 99.2% |
| Guyuan | TIGMIC166 | sheep | *D*. *silvarum* | *R*. *raoultii* (MN446747.2), 100% | *R*. *raoultii* (MN450413.2), 100% | Uncultured *Rickettsia* sp. (MG598409.1), 99.2% |
| Guyuan | TIGMIC167 | sheep | *D*. *silvarum* | *R*. *raoultii* (MN446747.2), 100% | *R*. *raoultii* (MN450413.2), 100% | Uncultured *Rickettsia* sp. (MN431836.1), 99.2% |
| Guyuan | TIGMIC168 | sheep | *D*. *silvarum* | *R*. *raoultii* (MN446747.2), 99.7% | *R*. *raoultii* (MK304548.1), 98.7% | Uncultured *Rickettsia* sp. (LC060714.1), 99.2% |
| Guyuan | TIGMIC173 | sheep | *D*. *nuttalli* | *R*. *sibirica* (MF098398.1), 100% | Uncultured *Rickettsia* sp. (MG598412.1), 100% | *R*. *aeschlimannii* (MK732478.1), 99.5% |
| Guyuan | TIGMIC174 | sheep | *D*. *nuttalli* | *R*. *sibirica* (MF098398.1), 100% | *Rickettsia* sp. (AF179365.1), 98.1% | *R*. *aeschlimannii* (MK732478.1), 99.5% |
| Guyuan | TIGMIC175 | sheep | *D*. *nuttalli* | *R*. *sibirica* (MF098398.1), 100% | Uncultured *Rickettsia* sp. (MG598412.1), 99.8% | *R*. *sibirica* (OM475658.1), 100% |
| Guyuan | TIGMIC176 | sheep | *D*. *nuttalli* | *R*. *sibirica* (MF098398.1), 100% | Uncultured *Rickettsia* sp. (MG598412.1), 100% | Uncultured *Rickettsia* sp. (MN431836.1), 99.7% |
| Guyuan | TIGMIC177 | sheep | *D*. *nuttalli* | *R*. *sibirica* (MF098398.1), 100% | Uncultured *Rickettsia* sp. (MG598412.1), 100% | *R*. *aeschlimannii* (MK732478.1), 99.5% |
| Guyuan | TIGMIC178 | sheep | *D*. *nuttalli* | *R*. *sibirica* (MF098398.1), 100% | Uncultured *Rickettsia* sp. (MG598412.1), 100% | *R*. *aeschlimannii* (MK732478.1), 99.7% |
| Guyuan | TIGMIC179 | sheep | *D*. *nuttalli* | *R*. *sibirica* (MF098398.1), 100% | Uncultured *Rickettsia* sp. (MG598412.1), 100% | *R*. *africae* (LC565701.1), 99.2% |
| Guyuan | TIGMIC180 | sheep | *D*. *nuttalli* | *R*. *sibirica* (MF098398.1), 100% | Uncultured *Rickettsia* sp. (MG598412.1), 100% | Uncultured *Rickettsia* sp. (MN431836.1), 99% |
| Guyuan | TIGMIC181 | sheep | *D*. *silvarum* | *R*. *slovaca* (MF002588.1), 100% | Uncultured *Rickettsia* sp. (MK181513.1), 100% | Uncultured *Rickettsia* sp. (MG598409.1), 99.5% |
| Guyuan | TIGMIC182 | sheep | *D*. *silvarum* | *R*. *slovaca* (MF002588.1), 100% | Uncultured *Rickettsia* sp. (MK181513.1), 99.8% | Uncultured *Rickettsia* sp. (MG598409.1), 99.7% |
| Guyuan | TIGMIC183 | NA | *D*. *nuttalli* | *R*. *slovaca* (MF002588.1), 100% | *R*. *slovaca* (MF379311.1), 99% | Uncultured *Rickettsia* sp. (MG598409.1), 99.7% |
| Guyuan | TIGMIC184 | NA | *D*. *nuttalli* | *R*. *slovaca* (MF002588.1), 100% | Uncultured *Rickettsia* sp. (MK181513.1), 100% | Uncultured *Rickettsia* sp. (MG598409.1), 99.7% |
| Guyuan | TIGMIC185 | NA | *D*. *silvarum* | *Ca*. R. jingxinensis (MH500194.1), 100% | *Ca*. R. jingxinensis (MN463682.1), 100% | *Ca*. R. jingxinensis (OR801782.1), 100% |
| Guyuan | TIGMIC186 | NA | *Hae*. *longicornis* | *Ca*. R. jingxinensis (MH500194.1), 100% | *Ca*. R. jingxinensis (MN463682.1), 100% | *Ca*. R. jingxinensis (OR801782.1), 100% |
| Guyuan | TIGMIC187 | NA | *Hae*. *longicornis* | *Ca*. R. jingxinensis (MH500194.1), 100% | *Ca*. R. jingxinensis (MN463682.1), 100% | *Ca*. R. jingxinensis (OR801782.1), 99.5% |
| Guyuan | TIGMIC188 | NA | *D*. *silvarum* | *Ca*. R. jingxinensis (MH500194.1), 100% | *Ca*. R. jingxinensis (MN463682.1), 100% | *Ca*. R. jingxinensis (OR801782.1), 99.7% |
| Guyuan | TIGMIC189 | NA | *Hae*. *longicornis* | *Ca*. R. jingxinensis (MH500194.1), 100% | *Ca*. R. jingxinensis (MN463682.1), 100% | *Ca*. R. jingxinensis (OR801782.1), 100% |
| Guyuan | TIGMIC190 | NA | *Hae*. *longicornis* | *Ca*. R. jingxinensis (MH500194.1), 100% | *Ca*. R. jingxinensis (MN463682.1), 100% | *Ca*. R. jingxinensis (OR801782.1), 99.5% |
| Guyuan | TIGMIC191 | NA | *Hae*. *longicornis* | *Ca*. R. jingxinensis (MH500194.1), 100% | *Ca*. R. jingxinensis (MN463682.1), 100% | *Ca*. R. jingxinensis (OR801782.1), 100% |
| Guyuan | TIGMIC192 | NA | *Hae*. *longicornis* | *Ca*. R. jingxinensis (MH500194.1), 100% | *Ca*. R. jingxinensis (MN463682.1), 100% | *Ca*. R. jingxinensis (OR801782.1), 100% |
| Guyuan | TIGMIC193 | NA | *Hae*. *longicornis* | *Ca*. R. jingxinensis (MH500194.1), 100% | *Ca*. R. jingxinensis (MN463682.1), 100% | *Ca*. R. jingxinensis (OR801782.1), 100% |
| Guyuan | TIGMIC194 | NA | *Hae*. *longicornis* | *Ca*. R. jingxinensis (MH500194.1), 100% | *Ca*. R. jingxinensis (MN463682.1), 100% | *Ca*. R. jingxinensis (OR801782.1), 100% |
| Guyuan | TIGMIC195 | sheep | *Hae*. *qinghaiensis* | *Ca*. R. jingxinensis (MH500194.1), 100% | *Ca*. R. jingxinensis (MN463682.1), 100% | *Ca*. R. jingxinensis (MW114883.1), 99.2% |
| Guyuan | TIGMIC207 | NA | *Hae*. *concinna* | *R*. *aeschlimannii* (MH923218.1), 99.8% | *Ca*. R. hongyuanensis (OL335948.1), 98.6% | Uncultured *Rickettsia* sp. (MN431834.1), 100% |
| Guyuan | TIGMIC208 | NA | *Hae*. *longicornis* | *Ca*. R. longicornii (MT535574.1), 100% | *Ca*. R. hongyuanensis (OL335948.1), 98.7% | Uncultured *Rickettsia* sp. (OR971697.1), 99.7% |
| Guyuan | TIGMIC209 | sheep | *Hae*. *japonica* | *Ca*. R. hongyuanensis (OK662395.1), 100% | *Ca*. R. principis (OP382385.1), 99.6% | Uncultured *Rickettsia* sp. (OR971697.1), 99.2% |
| Guyuan | TIGMIC210 | NA | *Hae*. *longicornis* | *Ca*. R. hongyuanensis (OK662395.1), 100% | *Ca*. R. hongyuanensis (OL335948.1), 98.6% | *Ca*. R. principis (OR805124.1), 100% |
| Shizuishan | TIGMIC012 | goat | *Hya*. *scupense* | *R*. *aeschlimannii* (MH923218.1), 99.9% | *R*. *aeschlimannii* (KX227782.1), 100% | *R*. *aeschlimannii* (OR687096.1), 99.5% |
| Shizuishan | TIGMIC013 | goat | *Hya*. *scupense* | *R*. *aeschlimannii* (MH923218.1), 100% | *R*. *aeschlimannii* (KX227782.1), 100% | *R*. *aeschlimannii* (OR687096.1), 99.5% |
| Shizuishan | TIGMIC014 | goat | *Hya*. *scupense* | *R*. *aeschlimannii* (MH923218.1), 100% | *R*. *aeschlimannii* (KX227782.1), 100% | *R*. *aeschlimannii* (OR687096.1), 99.7% |
| Shizuishan | TIGMIC015 | goat | *Hya*. *scupense* | *R*. *aeschlimannii* (MH923218.1), 100% | *R*. *aeschlimannii* (LC565690.1), 99.2% | *R*. *aeschlimannii* (OR687096.1), 99.5% |
| Shizuishan | TIGMIC016 | goat | *Hya*. *scupense* | *R*. *aeschlimannii* (MH923218.1), 100% | *R*. *aeschlimannii* (KX227782.1), 100% | *R*. *aeschlimannii* (OR687096.1), 99% |
| Shizuishan | TIGMIC017 | goat | *Hya*. *scupense* | *R*. *aeschlimannii* (MH923218.1), 100% | *R*. *aeschlimannii* (LC565690.1), 99.2% | *R*. *aeschlimannii* (OR687096.1), 99.5% |
| Shizuishan | TIGMIC018 | goat | *Hya*. *scupense* | *R*. *aeschlimannii* (MH923218.1), 100% | *R*. *aeschlimannii* (LC565690.1), 99.3% | *R*. *aeschlimannii* (OR687096.1), 99.5% |
| Shizuishan | TIGMIC019 | goat | *Hya*. *scupense* | *R*. *aeschlimannii* (MH923218.1), 99.9% | *R*. *aeschlimannii* (KX227782.1), 100% | Uncultured *Rickettsia* sp. (MN431836.1), 99.7% |
| Shizuishan | TIGMIC020 | goat | *Hya*. *scupense* | *R*. *aeschlimannii* (MH923218.1), 100% | *R*. *aeschlimannii* (LC565690.1), 99.5% | Uncultured *Rickettsia* sp. (MN431836.1), 99.7% |
| Shizuishan | TIGMIC021 | goat | *Hya*. *scupense* | *R*. *aeschlimannii* (MH923218.1), 100% | *R*. *aeschlimannii* (LC565690.1), 99.3% | *R*. *aeschlimannii* (OR687096.1), 99.2% |
| Shizuishan | TIGMIC022 | goat | *Hya*. *scupense* | *R*. *aeschlimannii* (MH923218.1), 100% | *R*. *aeschlimannii* (LC565690.1), 99.8% | *R*. *aeschlimannii* (OR687096.1), 99.2% |
| Shizuishan | TIGMIC023 | goat | *Hya*. *scupense* | *R*. *aeschlimannii* (MH923218.1), 99.9% | *R*. *aeschlimannii* (MG920564.1), 99.7% | *R*. *aeschlimannii* (MH675648.1), 99.7% |
| Shizuishan | TIGMIC024 | goat | *Hya*. *scupense* | *R*. *aeschlimannii* (MH923218.1), 100% | *R*. *aeschlimannii* (KX227782.1), 100% | *R*. *aeschlimannii* (OR687096.1), 99.5% |
| Shizuishan | TIGMIC025 | goat | *Hya*. *scupense* | *R*. *aeschlimannii* (MH923218.1), 99.9% | *R*. *aeschlimannii* (LC565690.1), 99.5% | Uncultured *Rickettsia* sp. (MN431836.1), 99.7% |
| Shizuishan | TIGMIC026 | goat | *Hya*. *scupense* | *R*. *aeschlimannii* (MH923218.1), 99.9% | *R*. *aeschlimannii* (KX227782.1), 100% | *R*. *aeschlimannii* (OR687096.1), 99.5% |
| Shizuishan | TIGMIC027 | goat | *Hya*. *scupense* | *R*. *aeschlimannii* (MH923218.1), 100% | *R*. *aeschlimannii* (KX227782.1), 100% | *R*. *aeschlimannii* (OR687096.1), 99.7% |
| Shizuishan | TIGMIC028 | goat | *Hya*. *scupense* | *R*. *aeschlimannii* (MH923218.1), 100% | *R*. *aeschlimannii* (LC565690.1), 99.5% | *R*. *aeschlimannii* (OR687096.1), 99.5% |
| Shizuishan | TIGMIC029 | goat | *Hya*. *scupense* | *R*. *aeschlimannii* (MH923218.1), 99.9% | *R*. *aeschlimannii* (LC565690.1), 99.7% | *R*. *aeschlimannii* (OR687096.1), 99.5% |
| Shizuishan | TIGMIC030 | goat | *Hya*. *scupense* | *R*. *aeschlimannii* (MH923218.1), 100% | *R*. *aeschlimannii* (KX227782.1), 100% | *R*. *aeschlimannii* (OR687096.1), 99.5% |
| Shizuishan | TIGMIC031 | goat | *Hya*. *scupense* | *R*. *aeschlimannii* (MH923218.1), 100% | *R*. *aeschlimannii* (KX227782.1), 99.6% | *R*. *aeschlimannii* (OR687096.1), 99.2% |
| Shizuishan | TIGMIC032 | goat | *Hya*. *scupense* | *R*. *aeschlimannii* (MH923218.1), 100% | *R*. *aeschlimannii* (KX227782.1), 100% | *R*. *aeschlimannii* (OR687096.1), 99.5% |
| Shizuishan | TIGMIC033 | goat | *Hya*. *scupense* | *R*. *aeschlimannii* (MH923218.1), 99.9% | *R*. *aeschlimannii* (KX227782.1), 100% | *R*. *aeschlimannii* (OR687096.1), 99.5% |
| Shizuishan | TIGMIC034 | goat | *Hya*. *scupense* | *R*. *aeschlimannii* (MH923218.1), 100% | *R*. *aeschlimannii* (KX227782.1), 100% | *R*. *aeschlimannii* (OR687096.1), 99.5% |
| Shizuishan | TIGMIC035 | goat | *Hya*. *scupense* | *R*. *aeschlimannii* (MH923218.1), 99.9% | *R*. *aeschlimannii* (KX227782.1), 100% | *R*. *aeschlimannii* (OR687096.1), 99.5% |
| Shizuishan | TIGMIC036 | goat | *Hya*. *scupense* | *R*. *aeschlimannii* (MH923218.1), 100% | *R*. *aeschlimannii* (LC565690.1), 99.3% | *R*. *aeschlimannii* (MH675648.1), 100% |
| Shizuishan | TIGMIC037 | goat | *Hya*. *scupense* | *R*. *aeschlimannii* (MH923218.1), 99.9% | *R*. *aeschlimannii* (KX227782.1), 100% | *R*. *aeschlimannii* (OR687096.1), 99.7% |
| Shizuishan | TIGMIC038 | goat | *Hya*. *scupense* | *R*. *aeschlimannii* (MH923218.1), 100% | *R*. *aeschlimannii* (KX227782.1), 100% | *R*. *aeschlimannii* (MH675648.1), 100% |
| Shizuishan | TIGMIC039 | goat | *Hya*. *scupense* | *R*. *aeschlimannii* (MH923218.1), 100% | *R*. *aeschlimannii* (KX227782.1), 100% | *R*. *aeschlimannii* (OR687096.1), 99.2% |
| Shizuishan | TIGMIC040 | goat | *Hya*. *scupense* | *R*. *aeschlimannii* (MH923218.1), 100% | *R*. *aeschlimannii* (LC565690.1), 99% | *R*. *aeschlimannii* (OR687096.1), 99.5% |
| Shizuishan | TIGMIC041 | goat | *Hya*. *scupense* | *R*. *aeschlimannii* (MH923218.1), 100% | *R*. *aeschlimannii* (KX227782.1), 100% | Uncultured *Rickettsia* sp. (LC060714.1), 99.5% |
| Shizuishan | TIGMIC042 | goat | *Hya*. *scupense* | *R*. *aeschlimannii* (MH923218.1), 99.9% | *R*. *aeschlimannii* (KX227782.1), 100% | *R*. *aeschlimannii* (MH675648.1), 99.7% |
| Shizuishan | TIGMIC043 | goat | *Hya*. *scupense* | *R*. *aeschlimannii* (MH923218.1), 100% | *R*. *aeschlimannii* (LC565690.1), 99.2% | *R*. *aeschlimannii* (MH675648.1), 100% |
| Shizuishan | TIGMIC044 | goat | *Hya*. *scupense* | *R*. *aeschlimannii* (MH923218.1), 100% | *R*. *aeschlimannii* (KX227782.1), 100% | *R*. *aeschlimannii* (MH675648.1), 99.7% |
| Shizuishan | TIGMIC045 | goat | *Hya*. *scupense* | *R*. *aeschlimannii* (MH923218.1), 100% | *R*. *aeschlimannii* (KX227782.1), 100% | Uncultured *Rickettsia* sp. (MN431836.1), 99.2% |
| Shizuishan | TIGMIC046 | goat | *Hya*. *scupense* | *R*. *aeschlimannii* (MH923218.1), 100% | *R*. *aeschlimannii* (LC565690.1), 99.3% | *R*. *aeschlimannii* (OR687096.1), 99.5% |
| Shizuishan | TIGMIC047 | goat | *Hya*. *scupense* | *R*. *aeschlimannii* (MH923218.1), 100% | *R*. *aeschlimannii* (KX227782.1), 100% | *R*. *aeschlimannii* (OR687096.1), 99.2% |
| Shizuishan | TIGMIC048 | goat | *Hya*. *scupense* | *R*. *aeschlimannii* (MH923218.1), 100% | *R*. *aeschlimannii* (LC565690.1), 99.2% | *R*. *aeschlimannii* (OR687096.1), 99.2% |
| Shizuishan | TIGMIC049 | goat | *Hya*. *scupense* | *R*. *aeschlimannii* (MH923218.1), 100% | *R*. *aeschlimannii* (LC565690.1), 99.5% | *R*. *aeschlimannii* (OR687096.1), 99.5% |
| Shizuishan | TIGMIC050 | goat | *Hya*. *scupense* | *R*. *aeschlimannii* (MH923218.1), 100% | *R*. *aeschlimannii* (KX227782.1), 100% | *R*. *aeschlimannii* (OR687096.1), 99.5% |
| Shizuishan | TIGMIC051 | goat | *Hya*. *scupense* | *R*. *aeschlimannii* (MH923218.1), 100% | *R*. *aeschlimannii* (KX227782.1), 100% | *R*. *aeschlimannii* (OR687096.1), 99.5% |
| Shizuishan | TIGMIC052 | goat | *Hya*. *scupense* | *R*. *aeschlimannii* (MH923218.1), 100% | *R*. *aeschlimannii* (LC565690.1), 99.5% | *R*. *aeschlimannii* (OR687096.1), 99.2% |
| Shizuishan | TIGMIC053 | goat | *Hya*. *scupense* | *R*. *aeschlimannii* (MH923218.1), 99.9% | *R*. *aeschlimannii* (LC565690.1), 99.5% | *R*. *aeschlimannii* (OR687096.1), 99.2% |
| Shizuishan | TIGMIC054 | goat | *Hya*. *scupense* | *R*. *aeschlimannii* (MH923218.1), 99.9% | *R*. *aeschlimannii* (KX227782.1), 100% | *R*. *aeschlimannii* (MH675648.1), 99.7% |
| Shizuishan | TIGMIC055 | goat | *Hya*. *scupense* | *R*. *aeschlimannii* (MH923218.1), 100% | *R*. *aeschlimannii* (LC565690.1), 99.7% | *R*. *aeschlimannii* (MH675648.1), 99.7% |
| Shizuishan | TIGMIC056 | goat | *Hya*. *scupense* | *R*. *aeschlimannii* (MH923218.1), 100% | *R*. *aeschlimannii* (KX227782.1), 100% | *R*. *aeschlimannii* (MH675648.1), 100% |
| Shizuishan | TIGMIC057 | goat | *Hya*. *scupense* | *R*. *aeschlimannii* (MH923218.1), 100% | *R*. *aeschlimannii* (KX227782.1), 100% | Uncultured *Rickettsia* sp. (MN431836.1), 99.2% |
| Shizuishan | TIGMIC058 | goat | *Hya*. *scupense* | *R*. *aeschlimannii* (MH923218.1), 100% | *R*. *aeschlimannii* (KX227782.1), 100% | *R*. *aeschlimannii* (MH675648.1), 100% |
| Shizuishan | TIGMIC059 | goat | *Hya*. *scupense* | *R*. *aeschlimannii* (MH923218.1), 100% | *R*. *aeschlimannii* (KX227782.1), 100% | *R*. *aeschlimannii* (OR687096.1), 99.7% |
| Shizuishan | TIGMIC060 | goat | *Hya*. *scupense* | *R*. *aeschlimannii* (MH923218.1), 100% | *R*. *aeschlimannii* (KX227782.1), 100% | *R*. *aeschlimannii* (OR687096.1), 99.7% |
| Shizuishan | TIGMIC061 | goat | *Hya*. *scupense* | *R*. *aeschlimannii* (MH923218.1), 100% | *R*. *aeschlimannii* (KX227782.1), 100% | *R*. *aeschlimannii* (OR687096.1), 99.5% |
| Shizuishan | TIGMIC062 | goat | *Hya*. *scupense* | *R*. *aeschlimannii* (MH923218.1), 100% | *R*. *aeschlimannii* (KX227782.1), 100% | *R*. *aeschlimannii* (OR687096.1), 99.7% |
| Shizuishan | TIGMIC063 | goat | *Hya*. *scupense* | *R*. *aeschlimannii* (MH923218.1), 100% | *R*. *aeschlimannii* (KX227782.1), 100% | *R*. *aeschlimannii* (OR687096.1), 100% |
| Shizuishan | TIGMIC064 | goat | *Hya*. *scupense* | *R*. *aeschlimannii* (MH923218.1), 100% | *R*. *aeschlimannii* (KX227782.1), 100% | *R*. *aeschlimannii* (OR687096.1), 99.5% |
| Wuzhong | TIGMIC070 | sheep | *D*. *nuttalli* | *R*. *raoultii* (MN446747.2), 100% | *R*. *raoultii* (MK304548.1), 99.5% | Uncultured *Rickettsia* sp. (MN431836.1), 100% |
| Wuzhong | TIGMIC071 | sheep | *D*. *nuttalli* | *R*. *raoultii* (ON191634.1), 100% | Uncultured *Rickettsia* sp. (MG598411.1), 99.8% | Uncultured *Rickettsia* sp. (MN431836.1), 99.5% |
| Wuzhong | TIGMIC072 | sheep | *D*. *nuttalli* | *R*. *raoultii* (MN446747.2), 100% | *R*. *raoultii* (MK304548.1), 99.5% | Uncultured *Rickettsia* sp. (MN431836.1), 99.7% |
| Wuzhong | TIGMIC073 | sheep | *D*. *nuttalli* | *R*. *raoultii* (MN446747.2), 100% | *R*. *raoultii* (MK304548.1), 99.7% | Uncultured *Rickettsia* sp. (MN431836.1), 99.5% |
| Wuzhong | TIGMIC074 | sheep | *D*. *nuttalli* | *R*. *raoultii* (ON191634.1), 100% | Uncultured *Rickettsia* sp. (MG598411.1), 100% | Uncultured *Rickettsia* sp. (LC060714.1), 99.7% |
| Wuzhong | TIGMIC075 | sheep | *D*. *nuttalli* | *R*. *raoultii* (MN446747.2), 100% | *R*. *raoultii* (MK304548.1), 99.5% | Uncultured *Rickettsia* sp. (LC060714.1), 99.7% |
| Wuzhong | TIGMIC076 | sheep | *D*. *nuttalli* | *R*. *raoultii* (MN446747.2), 99.6% | *R*. *raoultii* (MK304548.1), 100% | Uncultured *Rickettsia* sp. (LC060714.1), 99.7% |
| Wuzhong | TIGMIC077 | sheep | *D*. *nuttalli* | *R*. *raoultii* (MN446747.2), 100% | *R*. *raoultii* (MN450412.2), 100% | Uncultured *Rickettsia* sp. (LC060714.1), 99.7% |
| Wuzhong | TIGMIC078 | sheep | *D*. *nuttalli* | *R*. *raoultii* (ON191634.1), 100% | *R*. *raoultii* (MF511260.1), 99.3% | Uncultured *Rickettsia* sp. (MN431836.1), 99.7% |
| Wuzhong | TIGMIC079 | sheep | *D*. *nuttalli* | *R*. *raoultii* (ON191634.1), 100% | Uncultured *Rickettsia* sp. (MG598411.1), 100% | Uncultured *Rickettsia* sp. (LC060714.1), 99.5% |
| Wuzhong | TIGMIC080 | sheep | *D*. *nuttalli* | *R*. *raoultii* (MN446747.2), 100% | *R*. *raoultii* (MN450413.2), 100% | Uncultured *Rickettsia* sp. (MN431836.1), 99.7% |
| Wuzhong | TIGMIC081 | sheep | *D*. *nuttalli* | *R*. *raoultii* (MN446747.2), 100% | *R*. *raoultii* (MK304548.1), 99.7% | Uncultured *Rickettsia* sp. (MN431836.1), 99.5% |
| Wuzhong | TIGMIC082 | sheep | *D*. *nuttalli* | *R*. *raoultii* (MN446747.2), 100% | *R*. *raoultii* (MK304548.1), 99.1% | Uncultured *Rickettsia* sp. (LC060714.1), 99.7% |
| Wuzhong | TIGMIC083 | sheep | *D*. *nuttalli* | *R*. *raoultii* (MN446747.2), 99.7% | *R*. *raoultii* (MK304548.1), 99.3% | Uncultured *Rickettsia* sp. (MN431836.1), 100% |
| Wuzhong | TIGMIC084 | sheep | *D*. *nuttalli* | *R*. *raoultii* (MN446747.2), 100% | *R*. *raoultii* (MK304548.1), 99.5% | Uncultured *Rickettsia* sp. (MN431836.1), 100% |
| Wuzhong | TIGMIC085 | sheep | *D*. *nuttalli* | *R*. *raoultii* (ON191634.1), 100% | *R*. *raoultii* (MF511260.1), 99.5% | Uncultured *Rickettsia* sp. (MN431836.1), 99.5% |
| Wuzhong | TIGMIC086 | sheep | *D*. *nuttalli* | *R*. *raoultii* (MN446747.2), 100% | *R*. *raoultii* (MK304548.1), 99.4% | Uncultured *Rickettsia* sp. (MN431836.1), 100% |
| Wuzhong | TIGMIC087 | sheep | *D*. *nuttalli* | *R*. *raoultii* (MN446747.2), 100% | *R*. *raoultii* (MK304548.1), 99.7% | Uncultured *Rickettsia* sp. (MN431836.1), 99.7% |
| Wuzhong | TIGMIC088 | sheep | *D*. *nuttalli* | *R*. *raoultii* (MN446747.2), 100% | *R*. *raoultii* (MN450413.2), 100% | Uncultured *Rickettsia* sp. (LC060714.1), 99.7% |
| Wuzhong | TIGMIC090 | sheep | *D*. *nuttalli* | *R*. *raoultii* (MN446747.2), 99.9% | *R*. *raoultii* (MK304548.1), 100% | Uncultured *Rickettsia* sp. (LC060714.1), 99.7% |
| Wuzhong | TIGMIC091 | sheep | *D*. *nuttalli* | *R*. *raoultii* (MN446747.2), 99.9% | *R*. *raoultii* (MK304548.1), 99.3% | Uncultured *Rickettsia* sp. (LC060714.1), 99.7% |
| Wuzhong | TIGMIC092 | sheep | *D*. *nuttalli* | *R*. *raoultii* (MN446747.2), 100% | *R*. *raoultii* (MK304548.1), 99.5% | Uncultured *Rickettsia* sp. (MN431834.1), 100% |
| Wuzhong | TIGMIC093 | sheep | *D*. *nuttalli* | *R*. *raoultii* (MN446747.2), 100% | *R*. *raoultii* (MN450413.2), 100% | Uncultured *Rickettsia* sp. (MN431834.1), 100% |
| Wuzhong | TIGMIC094 | sheep | *D*. *nuttalli* | *R*. *raoultii* (MN446747.2), 100% | *R*. *raoultii* (MK304548.1), 99.6% | Uncultured *Rickettsia* sp. (LC060714.1), 99.7% |
| Wuzhong | TIGMIC095 | sheep | *D*. *nuttalli* | *R*. *raoultii* (MN446747.2), 100% | *R*. *raoultii* (MK304548.1), 99.8% | Uncultured *Rickettsia* sp. (MN431836.1), 99.7% |
| Wuzhong | TIGMIC096 | sheep | *D*. *nuttalli* | *R*. *raoultii* (MN446747.2), 100% | *R*. *raoultii* (MK304548.1), 99.4% | Uncultured *Rickettsia* sp. (MN431836.1), 100% |
| Wuzhong | TIGMIC097 | sheep | *D*. *nuttalli* | *R*. *raoultii* (MN446747.2), 100% | *R*. *raoultii* (MK304548.1), 99.5% | *R*. *africae* (LC565701.1), 99% |
| Wuzhong | TIGMIC098 | sheep | *D*. *nuttalli* | *R*. *raoultii* (MN446747.2), 99.7% | *R*. *raoultii* (MN450413.2), 100% | Uncultured *Rickettsia* sp. (MN431836.1), 99.5% |
| Wuzhong | TIGMIC099 | sheep | *D*. *nuttalli* | *R*. *raoultii* (MN446747.2), 100% | *R*. *raoultii* (MK304548.1), 99.7% | Uncultured *Rickettsia* sp. (LC060714.1), 100% |
| Wuzhong | TIGMIC100 | sheep | *D*. *nuttalli* | *R*. *raoultii* (MN446747.2), 99.9% | *R*. *raoultii* (MK304548.1), 99.5% | Uncultured *Rickettsia* sp. (MN431836.1), 100% |
| Wuzhong | TIGMIC101 | sheep | *D*. *nuttalli* | *R*. *raoultii* (MN446747.2), 100% | *R*. *raoultii* (MK304548.1), 99.8% | Uncultured *Rickettsia* sp. (MN431836.1), 99.7% |
| Wuzhong | TIGMIC102 | sheep | *D*. *nuttalli* | *R*. *raoultii* (MN446747.2), 100% | *R*. *raoultii* (MK304548.1), 98.1% | Uncultured *Rickettsia* sp. (MN431836.1), 100% |
| Wuzhong | TIGMIC103 | sheep | *D*. *nuttalli* | *R*. *raoultii* (MN446747.2), 99.9% | *R*. *raoultii* (MN450412.2), 100% | Uncultured *Rickettsia* sp. (MN431836.1), 99.5% |
| Wuzhong | TIGMIC104 | sheep | *D*. *nuttalli* | *R*. *raoultii* (MN446747.2), 100% | *R*. *raoultii* (MK304548.1), 99% | Uncultured *Rickettsia* sp. (MN431836.1), 99.5% |
| Wuzhong | TIGMIC105 | sheep | *D*. *nuttalli* | *R*. *raoultii* (MN446747.2), 100% | *R*. *raoultii* (MK304548.1), 98.8% | Uncultured *Rickettsia* sp. (MN431836.1), 100% |
| Wuzhong | TIGMIC106 | sheep | *D*. *nuttalli* | *R*. *raoultii* (MN446747.2), 100% | *R*. *raoultii* (MN450413.2), 100% | Uncultured *Rickettsia* sp. (MN431836.1), 100% |
| Wuzhong | TIGMIC144 | sheep | *D*. *nuttalli* | *R*. *raoultii* (MN446747.2), 100% | *R*. *raoultii* (MN450413.2), 100% | Uncultured *Rickettsia* sp. (MN431836.1), 99.2% |
| Wuzhong | TIGMIC145 | sheep | *D*. *nuttalli* | *R*. *raoultii* (ON191634.1), 100% | Uncultured *Rickettsia* sp. (MG598411.1), 100% | Uncultured *Rickettsia* sp. (MN431836.1), 99.2% |
| Wuzhong | TIGMIC146 | sheep | *D*. *nuttalli* | *R*. *raoultii* (MN446747.2), 100% | *R*. *raoultii* (MN450413.2), 100% | Uncultured *Rickettsia* sp. (LC060714.1), 99.2% |
| Wuzhong | TIGMIC147 | sheep | *D*. *nuttalli* | *R*. *raoultii* (MN446747.2), 99.9% | *R*. *raoultii* (MN450413.2), 100% | Uncultured *Rickettsia* sp. (LC060714.1), 100% |
| Wuzhong | TIGMIC148 | sheep | *D*. *nuttalli* | *R*. *raoultii* (MN446747.2), 100% | *R*. *raoultii* (MK304548.1), 99.2% | Uncultured *Rickettsia* sp. (MN431836.1), 99% |
| Wuzhong | TIGMIC149 | sheep | *D*. *nuttalli* | *R*. *raoultii* (MN446747.2), 100% | *R*. *raoultii* (MN450413.2), 100% | Uncultured *Rickettsia* sp. (MN431836.1), 99.2% |
| Wuzhong | TIGMIC150 | sheep | *D*. *nuttalli* | *R*. *raoultii* (MN446747.2), 100% | *R*. *raoultii* (MN450413.2), 100% | Uncultured *Rickettsia* sp. (MN431836.1), 100% |
| Wuzhong | TIGMIC151 | sheep | *D*. *nuttalli* | *R*. *raoultii* (MN446747.2), 100% | *R*. *raoultii* (MN450413.2), 100% | Uncultured *Rickettsia* sp. (LC060714.1), 99.2% |
| Wuzhong | TIGMIC152 | sheep | *D*. *nuttalli* | *R*. *raoultii* (MN446747.2), 99.7% | *R*. *raoultii* (MN450413.2), 100% | Uncultured *Rickettsia* sp. (MN431834.1), 100% |
| Wuzhong | TIGMIC153 | sheep | *D*. *nuttalli* | *R*. *raoultii* (MN446747.2), 100% | *R*. *raoultii* (MN450413.2), 100% | Uncultured *Rickettsia* sp. (LC060714.1), 99.7% |
| Wuzhong | TIGMIC154 | sheep | *D*. *nuttalli* | *R*. *raoultii* (MN446747.2), 99.7% | *R*. *raoultii* (MK304548.1), 98.4% | Uncultured *Rickettsia* sp. (LC060714.1), 99.7% |
| Wuzhong | TIGMIC155 | sheep | *D*. *nuttalli* | *R*. *raoultii* (MN446747.2), 100% | *R*. *raoultii* (MK304548.1), 99.7% | Uncultured *Rickettsia* sp. (MN431836.1), 100% |
| Wuzhong | TIGMIC156 | sheep | *D*. *nuttalli* | *R*. *raoultii* (MN446747.2), 99.7% | *R*. *raoultii* (MN450413.2), 100% | Uncultured *Rickettsia* sp. (LC060714.1), 99.5% |
| Wuzhong | TIGMIC169 | sheep | *D*. *nuttalli* | *R*. *sibirica* (MF098398.1), 100% | *Rickettsia* sp. (AF179365.1), 99.4% | *R*. *sibirica* (OM475658.1), 99.7% |
| Zhongwei | TIGMIC001 | sheep | *Hya*. *asiaticum* | *R*. *aeschlimannii* (MH923218.1), 100% | *R*. *aeschlimannii* (KX227782.1), 100% | Uncultured *Rickettsia* sp. (LC060714.1), 99.5% |
| Zhongwei | TIGMIC002 | sheep | *Hya*. *asiaticum* | *R*. *aeschlimannii* (MH923218.1), 100% | *R*. *aeschlimannii* (KX227782.1), 100% | Uncultured *Rickettsia* sp. (LC060714.1), 99.5% |
| Zhongwei | TIGMIC003 | sheep | *Hya*. *asiaticum* | *R*. *aeschlimannii* (MH923218.1), 100% | *R*. *aeschlimannii* (KX227782.1), 100% | Uncultured *Rickettsia* sp. (LC060714.1), 99% |
| Zhongwei | TIGMIC004 | sheep | *Hya*. *asiaticum* | *R*. *aeschlimannii* (MH923218.1), 100% | *R*. *aeschlimannii* (KX227782.1), 100% | Uncultured *Rickettsia* sp. (MN431836.1), 99.7% |
| Zhongwei | TIGMIC005 | sheep | *Hya*. *asiaticum* | *R*. *aeschlimannii* (MH923218.1), 100% | *R*. *aeschlimannii* (KX227782.1), 100% | *R*. *aeschlimannii* (OR687096.1), 99.5% |
| Zhongwei | TIGMIC006 | sheep | *Hya*. *asiaticum* | *R*. *aeschlimannii* (MH923218.1), 100% | *R*. *aeschlimannii* (KX227782.1), 99.8% | Uncultured *Rickettsia* sp. (LC060714.1), 99.5% |
| Zhongwei | TIGMIC007 | sheep | *Hya*. *asiaticum* | *R*. *aeschlimannii* (MH923218.1), 100% | *R*. *aeschlimannii* (KX227782.1), 100% | *R*. *aeschlimannii* (MH675648.1), 100% |
| Zhongwei | TIGMIC008 | sheep | *Hya*. *asiaticum* | *R*. *aeschlimannii* (MH923218.1), 99.7% | *R*. *aeschlimannii* (KX227782.1), 100% | *R*. *aeschlimannii* (OR687096.1), 99.5% |
| Zhongwei | TIGMIC009 | sheep | *Hya*. *asiaticum* | *R*. *aeschlimannii* (MH923218.1), 100% | *R*. *aeschlimannii* (KX227782.1), 100% | *R*. *aeschlimannii* (MH675648.1), 100% |
| Zhongwei | TIGMIC010 | sheep | *Hya*. *asiaticum* | *R*. *aeschlimannii* (MH923218.1), 100% | *R*. *aeschlimannii* (LC565690.1), 99% | *R*. *aeschlimannii* (MH675648.1), 100% |
| Zhongwei | TIGMIC011 | sheep | *Hya*. *asiaticum* | *R*. *aeschlimannii* (MH923218.1), 100% | *R*. *aeschlimannii* (KX227782.1), 100% | Uncultured *Rickettsia* sp. (LC060714.1), 99.5% |
| Zhongwei | TIGMIC065 | goat | *Hya*. *asiaticum* | *R*. *aeschlimannii* (MH923218.1), 100% | *R*. *aeschlimannii* (LC565690.1), 99.2% | *R*. *aeschlimannii* (MH675648.1), 100% |
| Zhongwei | TIGMIC119 | sheep | *D*. *nuttalli* | *R*. *raoultii* (MN446747.2), 100% | *R*. *raoultii* (MN450413.2), 100% | Uncultured *Rickettsia* sp. (LC060714.1), 100% |
| Zhongwei | TIGMIC120 | sheep | *D*. *nuttalli* | *R*. *raoultii* (MN446747.2), 100% | *R*. *raoultii* (MN450413.2), 100% | Uncultured *Rickettsia* sp. (MN431836.1), 100% |
| Zhongwei | TIGMIC121 | sheep | *D*. *nuttalli* | *R*. *raoultii* (MN446747.2), 100% | *R*. *raoultii* (MN450413.2), 100% | Uncultured *Rickettsia* sp. (LC060714.1), 100% |
| Zhongwei | TIGMIC122 | sheep | *D*. *nuttalli* | *R*. *raoultii* (MN446747.2), 100% | *R*. *raoultii* (MK304548.1), 99.3% | Uncultured *Rickettsia* sp. (MN431836.1), 100% |
| Zhongwei | TIGMIC123 | sheep | *D*. *nuttalli* | *R*. *raoultii* (MN446747.2), 100% | *R*. *raoultii* (MN450413.2), 100% | Uncultured *Rickettsia* sp. (LC060714.1), 100% |
| Zhongwei | TIGMIC124 | sheep | *D*. *nuttalli* | *R*. *raoultii* (MN446747.2), 100% | *R*. *raoultii* (MN450413.2), 100% | Uncultured *Rickettsia* sp. (MN431836.1), 100% |
| Zhongwei | TIGMIC125 | sheep | *D*. *nuttalli* | *R*. *sibirica* (MF098398.1), 99.9% | *R*. *raoultii* (MK304548.1), 98.3% | *R*. *raoultii* (KP742992.1), 99.7% |
| Zhongwei | TIGMIC126 | sheep | *D*. *nuttalli* | *R*. *raoultii* (MN446747.2), 99.7% | *R*. conorii (OR117557.1), 97.9% | Uncultured *Rickettsia* sp. (MN431836.1), 100% |
| Zhongwei | TIGMIC127 | sheep | *D*. *nuttalli* | *R*. *raoultii* (MN446747.2), 100% | *R*. *raoultii* (MN450413.2), 100% | Uncultured *Rickettsia* sp. (MN431836.1), 100% |
| Zhongwei | TIGMIC128 | sheep | *D*. *nuttalli* | *R*. *raoultii* (MN446747.2), 100% | *R*. *raoultii* (MN450413.2), 100% | Uncultured *Rickettsia* sp. (LC060714.1), 100% |
| Zhongwei | TIGMIC129 | sheep | *D*. *nuttalli* | *R*. *raoultii* (MN446747.2), 100% | *R*. *raoultii* (MN450413.2), 100% | Uncultured *Rickettsia* sp. (LC060714.1), 100% |
| Zhongwei | TIGMIC130 | sheep | *D*. *nuttalli* | *R*. *raoultii* (MN446747.2), 100% | *R*. *raoultii* (MK304548.1), 99.3% | Uncultured *Rickettsia* sp. (MN431836.1), 99.7% |
| Zhongwei | TIGMIC131 | sheep | *D*. *nuttalli* | *R*. *raoultii* (MN446747.2), 100% | *R*. *raoultii* (MN450413.2), 99.8% | Uncultured *Rickettsia* sp. (MN431836.1), 100% |
| Zhongwei | TIGMIC132 | sheep | *D*. *nuttalli* | *R*. *raoultii* (MT509815.1), 99.8% | *R*. *raoultii* (MK304548.1), 98.5% | Uncultured *Rickettsia* sp. (MN431836.1), 100% |
| Zhongwei | TIGMIC138 | sheep | *D*. *nuttalli* | *R*. *raoultii* (MN446747.2), 100% | *R*. *raoultii* (MN450413.2), 100% | Uncultured *Rickettsia* sp. (LC060714.1), 99.7% |
| Zhongwei | TIGMIC139 | sheep | *D*. *nuttalli* | *R*. *raoultii* (MN446747.2), 100% | *R*. *raoultii* (MN450413.2), 99.8% | Uncultured *Rickettsia* sp. (MN431836.1), 99.5% |
| Zhongwei | TIGMIC170 | sheep | *D*. *nuttalli* | *R*. *sibirica* (MF098398.1), 100% | *Rickettsia* sp. (AF179365.1), 99% | *R*. *sibirica* (OM475658.1), 100% |
| Zhongwei | TIGMIC171 | sheep | *D*. *nuttalli* | *R*. *sibirica* (MF098398.1), 100% | Uncultured *Rickettsia* sp. (MG598412.1), 100% | *R*. *sibirica* (OM475658.1), 100% |
| Zhongwei | TIGMIC172 | sheep | *D*. *nuttalli* | *R*. *raoultii* (ON191634.1), 100% | Uncultured *Rickettsia* sp. (MG598412.1), 99.8% | Uncultured *Rickettsia* sp. (MN431836.1), 99% |
| Zhongwei | TIGMIC196 | NA | *Ar*. *vulgaris* | *R*. *japonica* (CP047359.1), 100% | *R*. *vini* (MT062907.1), 99% | Uncultured *Rickettsia* sp. (LC060714.1), 99.2% |
| Zhongwei | TIGMIC197 | NA | *Ar*. *vulgaris* | *R*. *japonica* (CP047359.1), 100% | *R*. *vini* (MT062907.1), 99% | Uncultured *Rickettsia* sp. (MN431836.1), 99% |
| Zhongwei | TIGMIC198 | NA | *Ar*. *vulgaris* | *R*. *japonica* (CP047359.1), 100% | *R*. *vini* (MT062907.1), 99% | Uncultured *Rickettsia* sp. (MN431836.1), 99% |
| Zhongwei | TIGMIC199 | NA | *Ar*. *vulgaris* | *R*. *japonica* (CP047359.1), 100% | *R*. *vini* (MT062907.1), 99.4% | Uncultured *Rickettsia* sp. (LC060714.1), 99.2% |
| Zhongwei | TIGMIC200 | NA | *Ar*. *vulgaris* | *Rickettsia* sp. (MN577237.1), 98.8% | *Rickettsia* sp. (MW287618.1), 100% | Uncultured *Rickettsia* sp. (LC060714.1), 99.2% |
| Zhongwei | TIGMIC201 | NA | *Ar*. *vulgaris* | *Ca*. R. longicornii (MT535574.1), 99.9% | *Rickettsia* sp. (KF666477.1), 100% | Uncultured *Rickettsia* sp. (KY073144.1), 99.5% |
| Zhongwei | TIGMIC202 | NA | *Ar*. *vulgaris* | *R*. *japonica* (CP047359.1), 100% | *Rickettsia* sp. (KF666477.1), 100% | Uncultured *Rickettsia* sp. (KY659474.1), 99.7% |
| Zhongwei | TIGMIC203 | NA | *Ar*. *vulgaris* | *R*. *japonica* (CP047359.1), 100% | *R*. *vini* (MT062907.1), 99.4% | Uncultured *Rickettsia* sp. (MN431836.1), 98.9% |
| Zhongwei | TIGMIC204 | NA | *Ar*. *vulgaris* | *R*. *japonica* (CP047359.1), 100% | *Rickettsia* sp. (KF666477.1), 100% | Uncultured *Rickettsia* sp. (MN431836.1), 99.5% |
| Zhongwei | TIGMIC205 | NA | *Ar*. *vulgaris* | *Rickettsia* sp. (MH618379.1), 100% | *Rickettsia* sp. (MW287618.1), 100% | Uncultured *Rickettsia* sp. (LC060714.1), 99.2% |
| Zhongwei | TIGMIC206 | NA | *Ar*. *vulgaris* | *R*. *japonica* (CP047359.1), 100% | *R*. *vini* (MT062907.1), 99.2% | Uncultured *Rickettsia* sp. (LC060714.1), 98.7% |

NA = ticks from vegetation.
