# Supplementary material for: The first direct detection of spotted fever group Rickettsia spp. diversity in ticks from Ningxia, northwestern China
Source: PLoS Negl Trop Dis. 2025 Jan 2;19(1):e0012729. doi: 10.1371/journal.pntd.0012729 (PMC11695002; doi:10.1371/journal.pntd.0012729)
Supplement: S5 Table — (DOCX) [file pntd.0012729.s005.docx]

**S5 Table.** Sequences of *Rickettsia* and *Anaplasma* deposited in GenBank.

| Species | GenBank accession number | | | |
| --- | --- | --- | --- | --- |
|  | *rrs* | *gltA* | *ompA* | *groEL* |
| *R. raoultii* | PP110550, PP110551, PP110553, PP110568, PP110640–PP110666, PP110668–PP110677, PP110679, PP110680, PP110682–PP110684, PP110686, PP110688, PP110689,  PP110691–PP110693, PP110695–PP110700, PP110702–PP110705, PP110708–PP110713, PP110715–PP110719, PP110721, PP110725, PP110730, PP110731, PP110733–PP110757 | PP150191–PP150231, PP150233–PP150293 | PP117690, PP117691, PP117693, PP117708, PP117780–PP117806, PP117808–PP117817, PP117819, PP117820, PP117822–PP117824, PP117826, PP117828, PP117829,  PP117831–PP117833, PP117835–PP117840, PP117842–PP117845, PP117848–PP117853, PP117855–PP117859, PP117861, PP117865, PP117870, PP117871, PP117873–PP117897 | NA |
| *R. aeschlimannii* | PP110576–PP110639, PP110694 | PP150126–PP150190 | PP117716–PP117779, PP117834 | NA |
| *R. heilongjiangensis* | PP110678 | PP150232 | PP117818 | NA |
| *R. sibirica* | PP110667, PP110701, PP110706, PP110707, PP110720,  PP110722–PP110724, PP110726–PP110729 | PP150294–PP150305 | PP117807, PP117841, PP117846, PP117847, PP117860,  PP117862–PP117864, PP117866–PP117869 | NA |
| *R. slovaca* | PP110681, PP110685, PP110687, PP110690 | PP150306–PP150309 | PP117821, PP117825, PP117827, PP117830 | NA |
| *Ca.* R. hongyuanensis | PP110549, PP110575, PP110714, PP110758 | PP150332–PP150335 | PP117689, PP117715, PP117854, PP117898 | NA |
| *Ca.* R. jingxinensis | PP110552,  PP110554–PP110556, PP110569–PP110574, PP110732 | PP150310–PP150320 | PP117692,  PP117694–PP117696, PP117709–PP117714, PP117872 | NA |
| *Ca.* R. vulgarisii | PP110557–PP110567 | PP150321–PP150331 | PP117697–PP117707 | NA |
| *A. ovis* | PP106263–PP106360 | PP117094–PP117191 | NA | PP117399–PP117496 |

NA = Gene segment is not amplified in the *Rickettsia*/*Anaplasma* species.
