# Supplementary material for: The first direct detection of spotted fever group Rickettsia spp. diversity in ticks from Ningxia, northwestern China
Source: PLoS Negl Trop Dis. 2025 Jan 2;19(1):e0012729. doi: 10.1371/journal.pntd.0012729 (PMC11695002; doi:10.1371/journal.pntd.0012729)
Supplement: S6 Table — (DOCX) [file pntd.0012729.s006.docx]

**S6 Table**. **Positive sequence identity of detected *Anaplasma* spp**. **with BLAST analysis.**

| City | Sample number | Animal source | Tick source | Sequence homology results, % | | |
| --- | --- | --- | --- | --- | --- | --- |
|  |  |  |  | *rrs* | *groEL* | *gltA* |
| Guyuan | TIGMIC022 | NA | *D*. *nuttalli* | *A*. *ovis* (CP015994.2), 100% | *A*. *ovis* (KX579069.1), 99.8% | *A*. *ovis* (CP015994.2), 100% |
| Guyuan | TIGMIC023 | NA | *D*. *nuttalli* | *A*. *ovis* (CP015994.2), 100% | *A*. *ovis* (KX579069.1), 99.8% | *A*. *ovis* (CP015994.2), 100% |
| Guyuan | TIGMIC024 | NA | *D*. *nuttalli* | *A*. *ovis* (CP015994.2), 100% | *A*. *ovis* (OM648132.1), 99.9% | *A*. *ovis* (CP015994.2), 100% |
| Guyuan | TIGMIC025 | NA | *D*. *nuttalli* | *A*. *ovis* (CP015994.2), 100% | *A*. *ovis* (CP015994.2), 99.8% | *A*. *ovis* (CP015994.2), 100% |
| Guyuan | TIGMIC026 | NA | *D*. *nuttalli* | *A*. *ovis* (CP015994.2), 100% | *A*. *ovis* (KX579069.1), 100% | *A*. *ovis* (CP015994.2), 99.9% |
| Guyuan | TIGMIC027 | NA | *D*. *nuttalli* | *A*. *ovis* (KX579073.1), 100% | *A*. *ovis* (KX579069.1), 99.9% | *A*. *ovis* (CP015994.2), 100% |
| Guyuan | TIGMIC028 | NA | *D*. *nuttalli* | *A*. *ovis* (CP015994.2), 100% | *A*. *ovis* (CP015994.2), 99.8% | *Anaplasma* sp. (KX450266.1), 100% |
| Guyuan | TIGMIC029 | NA | *D*. *nuttalli* | *A*. *ovis* (CP015994.2), 100% | *A*. *ovis* (KX579069.1), 99.9% | *Anaplasma* sp. (KX450266.1), 100% |
| Guyuan | TIGMIC030 | NA | *D*. *nuttalli* | *A*. *ovis* (CP015994.2), 100% | *A*. *ovis* (KX579069.1), 100% | *A*. *ovis* (CP015994.2), 100% |
| Guyuan | TIGMIC031 | NA | *D*. *nuttalli* | *A*. *ovis* (CP015994.2), 100% | *A*. *ovis* (CP015994.2), 99.7% | *A*. *ovis* (CP015994.2), 100% |
| Guyuan | TIGMIC032 | NA | *D*. *nuttalli* | *A*. *ovis* (CP015994.2), 100% | *A*. *ovis* (KX579069.1), 100% | *A*. *ovis* (CP015994.2), 99.9% |
| Guyuan | TIGMIC054 | sheep | *D*. *nuttalli* | *A*. *ovis* (CP015994.2), 99.8% | *A*. *ovis* (OM648132.1), 100% | *A*. *capra* (OQ847089.1), 100% |
| Guyuan | TIGMIC055 | sheep | *D*. *nuttalli* | *A*. *ovis* (CP015994.2), 100% | *A*. *ovis* (CP015994.2), 99.9% | *A*. *capra* (OQ847089.1), 100% |
| Guyuan | TIGMIC056 | sheep | *D*. *nuttalli* | *A*. *ovis* (KX579073.1), 100% | *A*. *ovis* (OM648132.1), 100% | *Anaplasma* sp. (KX450266.1), 100% |
| Guyuan | TIGMIC057 | sheep | *D*. *nuttalli* | *A*. *ovis* (OR214930.1), 100% | *A*. *ovis* (OM648132.1), 100% | *Anaplasma* sp. (KX450266.1), 99.7% |
| Guyuan | TIGMIC058 | sheep | *D*. *nuttalli* | *A*. *ovis* (CP015994.2), 100% | *A*. *ovis* (CP015994.2), 99.7% | *A*. *capra* (OQ847089.1), 100% |
| Guyuan | TIGMIC059 | sheep | *D*. *nuttalli* | *A*. *ovis* (OR214930.1), 99.8% | *A*. *ovis* (OM648132.1), 100% | *Anaplasma* sp. (KX450266.1), 99.9% |
| Guyuan | TIGMIC060 | sheep | *D*. *nuttalli* | *A*. *ovis* (CP015994.2), 100% | *A*. *ovis* (CP015994.2), 99.8% | *Anaplasma* sp. (KX450266.1), 100% |
| Guyuan | TIGMIC061 | sheep | *D*. *nuttalli* | *A*. *ovis* (CP015994.2), 100% | *A*. *ovis* (CP015994.2), 99.7% | *Anaplasma* sp. (KX450266.1), 99.9% |
| Guyuan | TIGMIC062 | sheep | *D*. *nuttalli* | *A*. *ovis* (KX579073.1), 99.8% | *A*. *ovis* (OM648132.1), 99.9% | *A*. *capra* (OQ847089.1), 100% |
| Guyuan | TIGMIC063 | sheep | *D*. *nuttalli* | *A*. *ovis* (KX579073.1), 99.8% | *A*. *ovis* (CP015994.2), 99.9% | *Anaplasma* sp. (KX450266.1), 99.9% |
| Guyuan | TIGMIC064 | sheep | *D*. *nuttalli* | *A*. *ovis* (KX579073.1), 99.7% | *A*. *ovis* (KX579069.1), 100% | *A*. *capra* (OQ847089.1), 100% |
| Guyuan | TIGMIC071 | NA | *D*. *silvarum* | *A*. *ovis* (CP015994.2), 100% | *A*. *ovis* (KX579069.1), 100% | *Anaplasma* sp. (KX450266.1), 99.9% |
| Guyuan | TIGMIC072 | NA | *D*. *silvarum* | *A*. *ovis* (CP015994.2), 100% | *A*. *ovis* (CP015994.2), 99.9% | *A*. *capra* (OQ847089.1), 100% |
| Guyuan | TIGMIC073 | sheep | *D*. *silvarum* | *A*. *ovis* (CP015994.2), 100% | *A*. *ovis* (KX579069.1), 99.8% | *A*. *ovis* (CP015994.2), 100% |
| Guyuan | TIGMIC074 | sheep | *D*. *silvarum* | *A*. *ovis* (CP015994.2), 100% | *A*. *ovis* (CP015994.2), 99.8% | *A*. *ovis* (CP015994.2), 100% |
| Guyuan | TIGMIC075 | sheep | *D*. *silvarum* | *A*. *ovis* (OR214930.1), 99.8% | *A*. *ovis* (KX579069.1), 100% | *A*. *ovis* (CP015994.2), 99.9% |
| Guyuan | TIGMIC076 | sheep | *D*. *silvarum* | *A*. *ovis* (MG869525.1), 100% | *A*. *ovis* (AF441131.1), 100% | *Anaplasma* sp. (KX450266.1), 100% |
| Guyuan | TIGMIC077 | sheep | *D*. *silvarum* | *A*. *ovis* (MG869525.1), 100% | *A*. *ovis* (AF441131.1), 99.9% | *Anaplasma* sp. (KX450266.1), 100% |
| Guyuan | TIGMIC078 | sheep | *D*. *silvarum* | *A*. *ovis* (KX579073.1), 99.7% | *A*. *ovis* (KX579069.1), 99.7% | *A*. *ovis* (CP015994.2), 100% |
| Guyuan | TIGMIC079 | sheep | *D*. *silvarum* | *A*. *ovis* (MG869525.1), 100% | *A*. *ovis* (AF441131.1), 99.9% | *Anaplasma* sp. (KX450266.1), 100% |
| Guyuan | TIGMIC080 | sheep | *D*. *silvarum* | *A*. *ovis* (CP015994.2), 100% | *A*. *ovis* (AF441131.1), 99.9% | *A*. *ovis* (CP015994.2), 100% |
| Guyuan | TIGMIC081 | sheep | *D*. *silvarum* | *A*. *ovis* (KX579073.1), 99.7% | *A*. *ovis* (KX579069.1), 99.9% | *Anaplasma* sp. (KX450266.1), 100% |
| Guyuan | TIGMIC082 | sheep | *D*. *silvarum* | *A*. *ovis* (CP015994.2), 99.8% | *A*. *ovis* (KX579069.1), 99.9% | *A*. *capra* (OQ847089.1), 100% |
| Guyuan | TIGMIC083 | sheep | *D*. *silvarum* | *A*. *ovis* (KX579073.1), 99.7% | *A*. *ovis* (KX579069.1), 99.9% | *Anaplasma* sp. (KX450266.1), 100% |
| Guyuan | TIGMIC084 | sheep | *D*. *silvarum* | *A*. *ovis* (CP015994.2), 100% | *A*. *ovis* (CP015994.2), 99.7% | *A*. *capra* (OQ847089.1), 100% |
| Guyuan | TIGMIC085 | sheep | *D*. *silvarum* | *A*. *ovis* (CP015994.2), 100% | *A*. *ovis* (CP015994.2), 99.7% | *A*. *capra* (OQ847089.1), 100% |
| Guyuan | TIGMIC086 | sheep | *D*. *silvarum* | *A*. *ovis* (OR214930.1), 99.8% | *A*. *ovis* (KX579069.1), 99.9% | *A*. *capra* (OQ847089.1), 100% |
| Guyuan | TIGMIC087 | sheep | *D*. *silvarum* | *A*. *ovis* (CP015994.2), 99.8% | *A*. *ovis* (KX579069.1), 99.9% | *A*. *capra* (OQ847089.1), 100% |
| Guyuan | TIGMIC088 | sheep | *D*. *silvarum* | *A*. *ovis* (CP015994.2), 100% | *A*. *ovis* (KX579069.1), 99.9% | *A*. *capra* (OQ847089.1), 100% |
| Guyuan | TIGMIC089 | sheep | *D*. *silvarum* | *A*. *ovis* (CP015994.2), 99.8% | *A*. *ovis* (CP015994.2), 99.8% | *A*. *capra* (OQ847089.1), 100% |
| Guyuan | TIGMIC090 | sheep | *D*. *silvarum* | *A*. *ovis* (CP015994.2), 100% | *A*. *ovis* (CP015994.2), 99.7% | *A*. *capra* (OQ847089.1), 100% |
| Guyuan | TIGMIC091 | sheep | *D*. *silvarum* | Uncultured *Anaplasma* sp. (OR792789.1), 100% | *A*. *ovis* (KX579069.1), 100% | *A*. *capra* (OQ847089.1), 100% |
| Guyuan | TIGMIC092 | sheep | *D*. *silvarum* | *A*. *ovis* (JQ917900.1), 100% | *A*. *ovis* (KX579069.1), 100% | *A*. *capra* (OQ847089.1), 100% |
| Guyuan | TIGMIC093 | sheep | *D*. *silvarum* | Uncultured *Anaplasma* sp. (OR792789.1), 100% | *A*. *ovis* (KX579069.1), 100% | *A*. *capra* (OQ847089.1), 100% |
| Guyuan | TIGMIC094 | sheep | *D*. *silvarum* | *A*. *ovis* (CP015994.2), 100% | *A*. *ovis* (KX579069.1), 100% | *A*. *capra* (OQ847089.1), 100% |
| Guyuan | TIGMIC095 | sheep | *D*. *silvarum* | *A*. *ovis* (CP015994.2), 100% | *A*. *ovis* (KX579069.1), 100% | *A*. *capra* (OQ847089.1), 99.9% |
| Guyuan | TIGMIC096 | NA | *Hae*. *longicornis* | *A*. *ovis* (OR214930.1), 99.8% | *A*. *ovis* (KX579069.1), 99.9% | *A*. *capra* (OQ847089.1), 99.9% |
| Guyuan | TIGMIC097 | NA | *Hae*. *longicornis* | Uncultured *Anaplasma* sp. (JN715833.1), 99.5% | *A*. *ovis* (KX579069.1), 99.8% | *A*. *capra* (OQ847089.1), 99.9% |
| Guyuan | TIGMIC098 | sheep | *Hae*. *qinghaiensis* | Uncultured *Anaplasma* sp. (OR792787.1), 99.8% | *A*. *ovis* (CP015994.2), 99.9% | *A*. *capra* (OQ847085.1), 100% |
| Wuzhong | TIGMIC001 | sheep | *D*. *nuttalli* | *A*. *ovis* (CP015994.2), 100% | *A*. *ovis* (CP015994.2), 99.8% | *A*. *capra* (OQ847088.1), 100% |
| Wuzhong | TIGMIC002 | sheep | *D*. *nuttalli* | *A*. *ovis* (CP015994.2), 100% | *A*. *ovis* (MG869402.1), 99.8% | *Anaplasma* sp. (KX450266.1), 100% |
| Wuzhong | TIGMIC003 | sheep | *D*. *nuttalli* | *A*. *ovis* (CP015994.2), 100% | *A*. *ovis* (KX579069.1), 100% | *A*. *ovis* (CP015994.2), 100% |
| Wuzhong | TIGMIC004 | sheep | *D*. *nuttalli* | *A*. *ovis* (CP015994.2), 100% | *A*. *ovis* (KX579069.1), 99.9% | *A*. *ovis* (CP015994.2), 100% |
| Wuzhong | TIGMIC005 | sheep | *D*. *nuttalli* | *A*. *ovis* (CP015994.2), 99.8% | *A*. *ovis* (OM648132.1), 100% | *A*. *ovis* (CP015994.2), 100% |
| Wuzhong | TIGMIC006 | sheep | *D*. *nuttalli* | *A*. *ovis* (CP015994.2), 100% | *A*. *ovis* (CP015994.2), 99.8% | *A*. *ovis* (CP015994.2), 99.9% |
| Wuzhong | TIGMIC007 | sheep | *D*. *nuttalli* | *A*. *ovis* (CP015994.2), 99.8% | *A*. *ovis* (KX579069.1), 100% | *A*. *ovis* (CP015994.2), 100% |
| Wuzhong | TIGMIC008 | sheep | *D*. *nuttalli* | *A*. *ovis* (CP015994.2), 100% | *A*. *ovis* (CP015994.2), 99.8% | *A*. *ovis* (CP015994.2), 100% |
| Wuzhong | TIGMIC009 | sheep | *D*. *nuttalli* | *A*. *ovis* (KX579073.1), 99.8% | *A*. *ovis* (KX579069.1), 99.9% | *A*. *ovis* (CP015994.2), 99.9% |
| Wuzhong | TIGMIC010 | sheep | *D*. *nuttalli* | *A*. *ovis* (CP015994.2), 100% | *A*. *ovis* (OM648132.1), 100% | *A*. *ovis* (CP015994.2), 99.7% |
| Wuzhong | TIGMIC011 | sheep | *D*. *nuttalli* | *A*. *ovis* (KX579073.1), 99.8% | *A*. *ovis* (AF441131.1), 100% | *A*. *ovis* (CP015994.2), 100% |
| Wuzhong | TIGMIC012 | sheep | *D*. *nuttalli* | *A*. *ovis* (CP015994.2), 100% | *A*. *ovis* (OM648132.1), 99.9% | *A*. *ovis* (CP015994.2), 100% |
| Wuzhong | TIGMIC013 | sheep | *D*. *nuttalli* | *A*. *ovis* (CP015994.2), 100% | *A*. *ovis* (AF441131.1), 100% | *A*. *ovis* (CP015994.2), 100% |
| Wuzhong | TIGMIC014 | sheep | *D*. *nuttalli* | *A*. *ovis* (KX579073.1), 100% | *A*. *ovis* (KX579069.1), 99.8% | *A*. *ovis* (CP015994.2), 100% |
| Wuzhong | TIGMIC015 | sheep | *D*. *nuttalli* | *A*. *ovis* (CP015994.2), 100% | *A*. *ovis* (KX579069.1), 100% | *A*. *ovis* (CP015994.2), 100% |
| Wuzhong | TIGMIC016 | sheep | *D*. *nuttalli* | *A*. *ovis* (CP015994.2), 100% | *A*. *ovis* (OM648132.1), 100% | *A*. *ovis* (CP015994.2), 100% |
| Wuzhong | TIGMIC017 | sheep | *D*. *nuttalli* | *A*. *ovis* (CP015994.2), 100% | *A*. *ovis* (CP015994.2), 99.6% | *A*. *ovis* (CP015994.2), 100% |
| Wuzhong | TIGMIC018 | sheep | *D*. *nuttalli* | *A*. *ovis* (CP015994.2), 100% | *A*. *ovis* (KX579069.1), 99.9% | *A*. *ovis* (CP015994.2), 100% |
| Wuzhong | TIGMIC019 | sheep | *D*. *nuttalli* | *A*. *ovis* (CP015994.2), 100% | *A*. *ovis* (AF441131.1), 100% | *A*. *ovis* (CP015994.2), 100% |
| Wuzhong | TIGMIC020 | sheep | *D*. *nuttalli* | *A*. *ovis* (CP015994.2), 100% | *A*. *ovis* (KX579069.1), 100% | *A*. *ovis* (CP015994.2), 100% |
| Wuzhong | TIGMIC021 | sheep | *D*. *nuttalli* | *A*. *ovis* (CP015994.2), 100% | *A*. *ovis* (KX579069.1), 99.9% | *A*. *ovis* (CP015994.2), 100% |
| Wuzhong | TIGMIC065 | sheep | *D*. *nuttalli* | *A*. *ovis* (KX579073.1), 100% | *A*. *ovis* (CP015994.2), 99.7% | *Anaplasma* sp. (KX450266.1), 99.9% |
| Wuzhong | TIGMIC066 | sheep | *D*. *nuttalli* | *A*. *ovis* (CP015994.2), 100% | *A*. *ovis* (AF441131.1), 100% | *A*. *capra* (OQ847089.1), 100% |
| Wuzhong | TIGMIC067 | sheep | *D*. *nuttalli* | *A*. *ovis* (KX579073.1), 100% | *A*. *ovis* (KX579069.1), 100% | *A*. *capra* (OQ847089.1), 100% |
| Wuzhong | TIGMIC068 | sheep | *D*. *nuttalli* | *A*. *ovis* (JQ917900.1), 100% | *A*. *ovis* (AF441131.1), 100% | *A*. *capra* (OQ847089.1), 100% |
| Wuzhong | TIGMIC069 | sheep | *D*. *nuttalli* | *A*. *ovis* (CP015994.2), 100% | *A*. *ovis* (KX579069.1), 100% | *A*. *capra* (OQ847089.1), 100% |
| Wuzhong | TIGMIC070 | sheep | *D*. *nuttalli* | *A*. *ovis* (CP015994.2), 100% | *A*. *ovis* (KX579069.1), 100% | *A*. *capra* (OQ847089.1), 100% |
| Zhongwei | TIGMIC033 | sheep | *D*. *nuttalli* | *A*. *ovis* (CP015994.2), 100% | *A*. *ovis* (CP015994.2), 99.8% | *Anaplasma* sp. (KX450266.1), 99.9% |
| Zhongwei | TIGMIC034 | sheep | *D*. *nuttalli* | *A*. *ovis* (CP015994.2), 100% | *A*. *ovis* (CP015994.2), 99.8% | *Anaplasma* sp. (KX450266.1), 100% |
| Zhongwei | TIGMIC035 | sheep | *D*. *nuttalli* | *A*. *ovis* (CP015994.2), 100% | *A*. *ovis* (CP015994.2), 99.8% | *A*. *capra* (MZ130264.1), 100% |
| Zhongwei | TIGMIC036 | sheep | *D*. *nuttalli* | *A*. *ovis* (CP015994.2), 100% | *A*. *ovis* (CP015994.2), 99.8% | *A*. *capra* (OQ847089.1), 100% |
| Zhongwei | TIGMIC037 | sheep | *D*. *nuttalli* | *A*. *ovis* (MG869525.1), 100% | *A*. *ovis* (KX579069.1), 99.9% | *Anaplasma* sp. (KX450266.1), 100% |
| Zhongwei | TIGMIC038 | sheep | *D*. *nuttalli* | *A*. *ovis* (CP015994.2), 100% | *A*. *ovis* (CP015994.2), 99.8% | *Anaplasma* sp. (KX450266.1), 99.7% |
| Zhongwei | TIGMIC039 | sheep | *D*. *nuttalli* | *A*. *ovis* (KX579073.1), 100% | *A*. *ovis* (KX579069.1), 100% | *A*. *capra* (OQ847089.1), 100% |
| Zhongwei | TIGMIC040 | sheep | *D*. *nuttalli* | *A*. *ovis* (CP015994.2), 100% | *A*. *ovis* (KX579069.1), 99.9% | *A*. *capra* (OQ847089.1), 100% |
| Zhongwei | TIGMIC041 | sheep | *D*. *nuttalli* | *A*. *ovis* (CP015994.2), 100% | *A*. *ovis* (KX579069.1), 100% | *Anaplasma* sp. (KX450266.1), 99.9% |
| Zhongwei | TIGMIC042 | sheep | *D*. *nuttalli* | *A*. *ovis* (CP015994.2), 100% | *A*. *ovis* (CP015994.2), 99.7% | *Anaplasma* sp. (KX450266.1), 99.9% |
| Zhongwei | TIGMIC043 | sheep | *D*. *nuttalli* | *A*. *ovis* (OR214930.1), 99.8% | *A*. *ovis* (KX579069.1), 100% | *A*. *capra* (OQ847089.1), 99.9% |
| Zhongwei | TIGMIC044 | sheep | *D*. *nuttalli* | *A*. *ovis* (CP015994.2), 100% | *A*. *ovis* (CP015994.2), 99.7% | *A*. *capra* (OQ847089.1), 100% |
| Zhongwei | TIGMIC045 | sheep | *D*. *nuttalli* | *A*. *ovis* (CP015994.2), 100% | *A*. *ovis* (KX579069.1), 100% | *A*. *capra* (OQ847089.1), 100% |
| Zhongwei | TIGMIC046 | sheep | *D*. *nuttalli* | *A*. *ovis* (OR214930.1), 99.7% | *A*. *ovis* (CP015994.2), 99.8% | *A*. *capra* (OQ847089.1), 100% |
| Zhongwei | TIGMIC047 | sheep | *D*. *nuttalli* | *A*. *ovis* (CP015994.2), 100% | *A*. *ovis* (CP015994.2), 99.9% | *A*. *capra* (OQ847089.1), 100% |
| Zhongwei | TIGMIC048 | sheep | *D*. *nuttalli* | *A*. *ovis* (CP015994.2), 100% | *A*. *ovis* (CP015994.2), 99.9% | *A*. *capra* (OQ847089.1), 100% |
| Zhongwei | TIGMIC049 | sheep | *D*. *nuttalli* | *A*. *ovis* (CP015994.2), 100% | *A*. *ovis* (CP015994.2), 99.9% | *A*. *capra* (OQ847089.1), 100% |
| Zhongwei | TIGMIC050 | sheep | *D*. *nuttalli* | *A*. *ovis* (KX579073.1), 100% | *A*. *ovis* (KX579069.1), 100% | *Anaplasma* sp. (KX450266.1), 99.9% |
| Zhongwei | TIGMIC051 | sheep | *D*. *nuttalli* | *A*. *ovis* (KX579073.1), 100% | *A*. *ovis* (OM648132.1), 100% | *A*. *capra* (OQ847089.1), 100% |
| Zhongwei | TIGMIC052 | sheep | *D*. *nuttalli* | *A*. *ovis* (CP015994.2), 100% | *A*. *ovis* (KX579069.1), 99.8% | *A*. *capra* (OQ847089.1), 100% |
| Zhongwei | TIGMIC053 | sheep | *D*. *nuttalli* | *A*. *ovis* (KX579073.1), 100% | *A*. *ovis* (OM648132.1), 99.9% | *A*. *capra* (OQ847089.1), 100% |

NA = ticks from vegetation.
