# Supplementary material for: The first direct detection of spotted fever group Rickettsia spp. diversity in ticks from Ningxia, northwestern China
Source: PLoS Negl Trop Dis. 2025 Jan 2;19(1):e0012729. doi: 10.1371/journal.pntd.0012729 (PMC11695002; doi:10.1371/journal.pntd.0012729)
Supplement: S7 Table — (DOCX) [file pntd.0012729.s007.docx]

**S7 Table.** **Prevalence of rickettsiae and *Anaplasma ovis* in ticks from different cities of Ningxia.**

| City | *D. nuttalli* | *D. silvarum* | *Hya. asiaticum* | *Hya. scupense* | *Hae. concinna* | *Hae. japonica* | *Hae. longicornis* | *Hae. qinghaiensis* | *Ar. vulgaris* | Total |
| --- | --- | --- | --- | --- | --- | --- | --- | --- | --- | --- |
| Guyuan | 18 (66.7%)*, 22 (81.5%)** | 29 (44.6%), 25 (38.5%) | 0 | 0 | 1 (10.0%), 0 | 3 (8.33%), 0 | 11 (26.2%), 2 (4.8%) | 3 (8.3%), 1 (2.8%) | 0 | 65 (23.4%), 50(19.5%) |
| Shizuishan | 0 | 0 | 0 | 53 (68.8%), 0 | 0 | 0 | 0 | 0 | 0 | 53 (68.8%), 0 |
| Wuzhong | 50 (74.6%), 27 (40.3%) | 0 | 0 | 0 | 0 | 0 | 0 | 0 | 0 | 50 (74.6%), 27 (40.3%) |
| Zhongwei | 19 (70.4%), 21 (77.8%) | 0 | 12 (50.0%), 0 | 0 | 0 | 0 | 0 | 0 | 11 (78.6%), 0 | 42 (64.6%), 21 (32.3%) |
| Total | 87 (71.9%), 70 (57.9%) | 29 (44.6%), 25 (38.5%) | 12 (50.0%), 0 | 53 (68.8%), 0 | 1 (10.0%), 0 | 3 (8.33%), 0 | 11 (26.2%), 2 (4.8%) | 3 (8.3%), 1 (2.8%) | 11 (78.6%), 0 | 210 (49.4%), 98 (22.2%) |

* = number and positive rate of rickettsiae in tick species;

** = number and positive rate of *Anaplasma ovis* in tick species.
