# Supplementary figures and images for: The first direct detection of spotted fever group Rickettsia spp. diversity in ticks from Ningxia, northwestern China
Source: PLoS Negl Trop Dis. 2025 Jan 2;19(1):e0012729. doi: 10.1371/journal.pntd.0012729 (PMC11695002; doi:10.1371/journal.pntd.0012729)

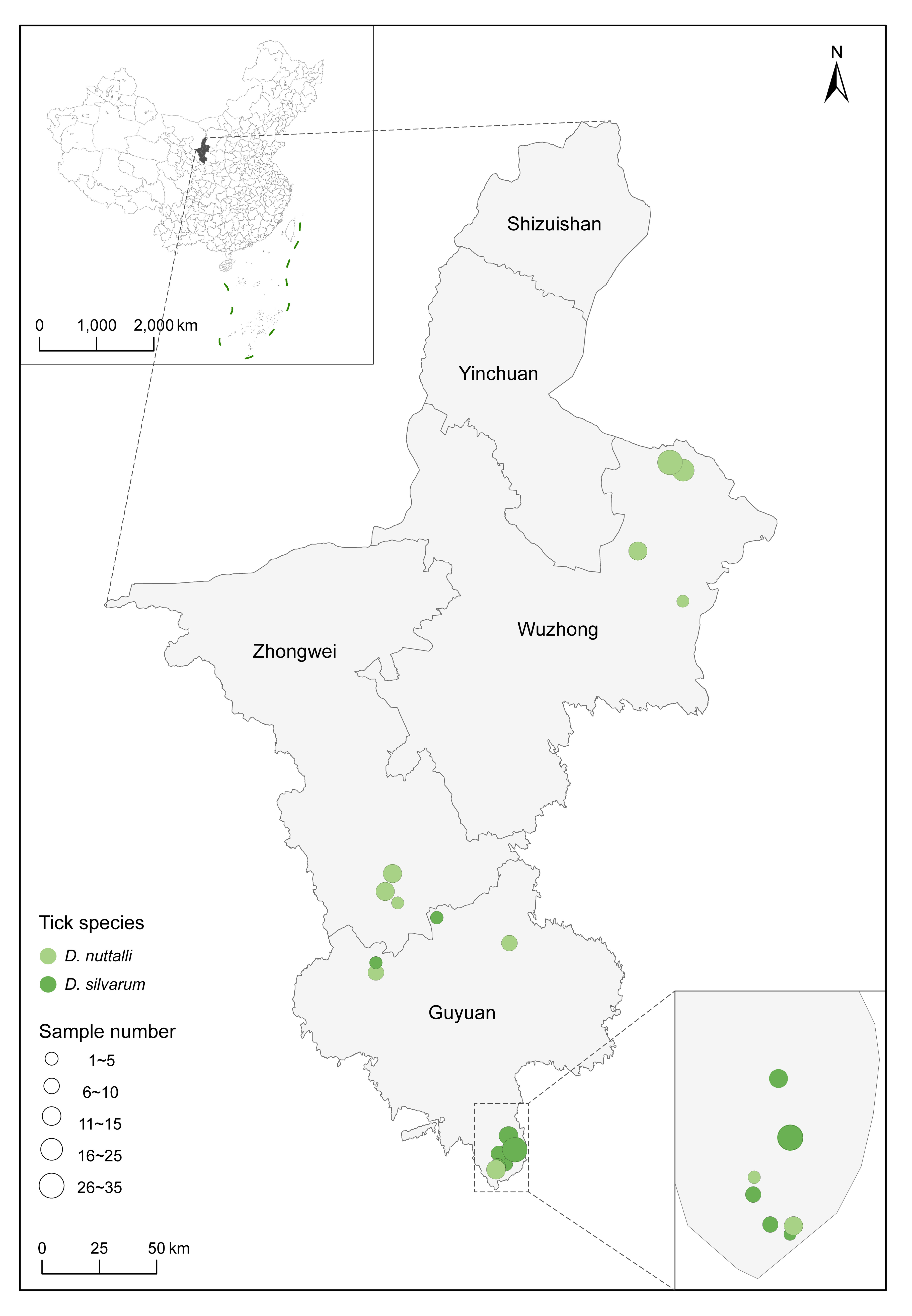

Supplement: S1 Fig — Different colour and size of circles represent the species and number of ticks collected from Ningxia. The map was constructed using ArcGIS v10.8.2 software. The basemap shapefiles were downloaded from the Chinese Resource and Environmental Science Data Platform (http://www.resdc.cn/, DOI:10.12078/2023010102). (TIF) [file pntd.0012729.s008.tif]

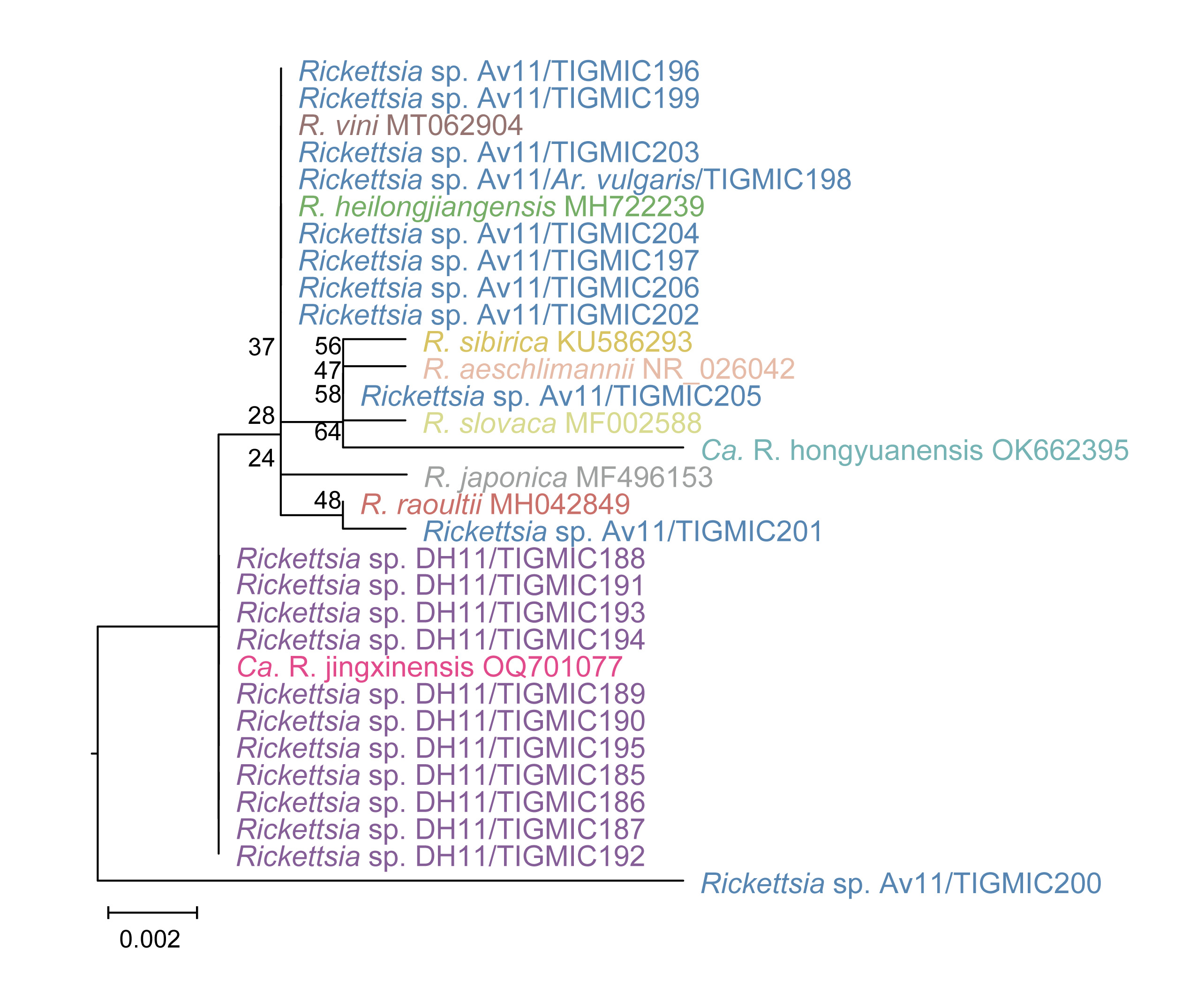

Supplement: S2 Fig — Numbers at the nodes are bootstrap proportions with 1000 replicates. The scale bar indicates the number of nucleotide substitutions per site. (TIF) [file pntd.0012729.s009.tif]

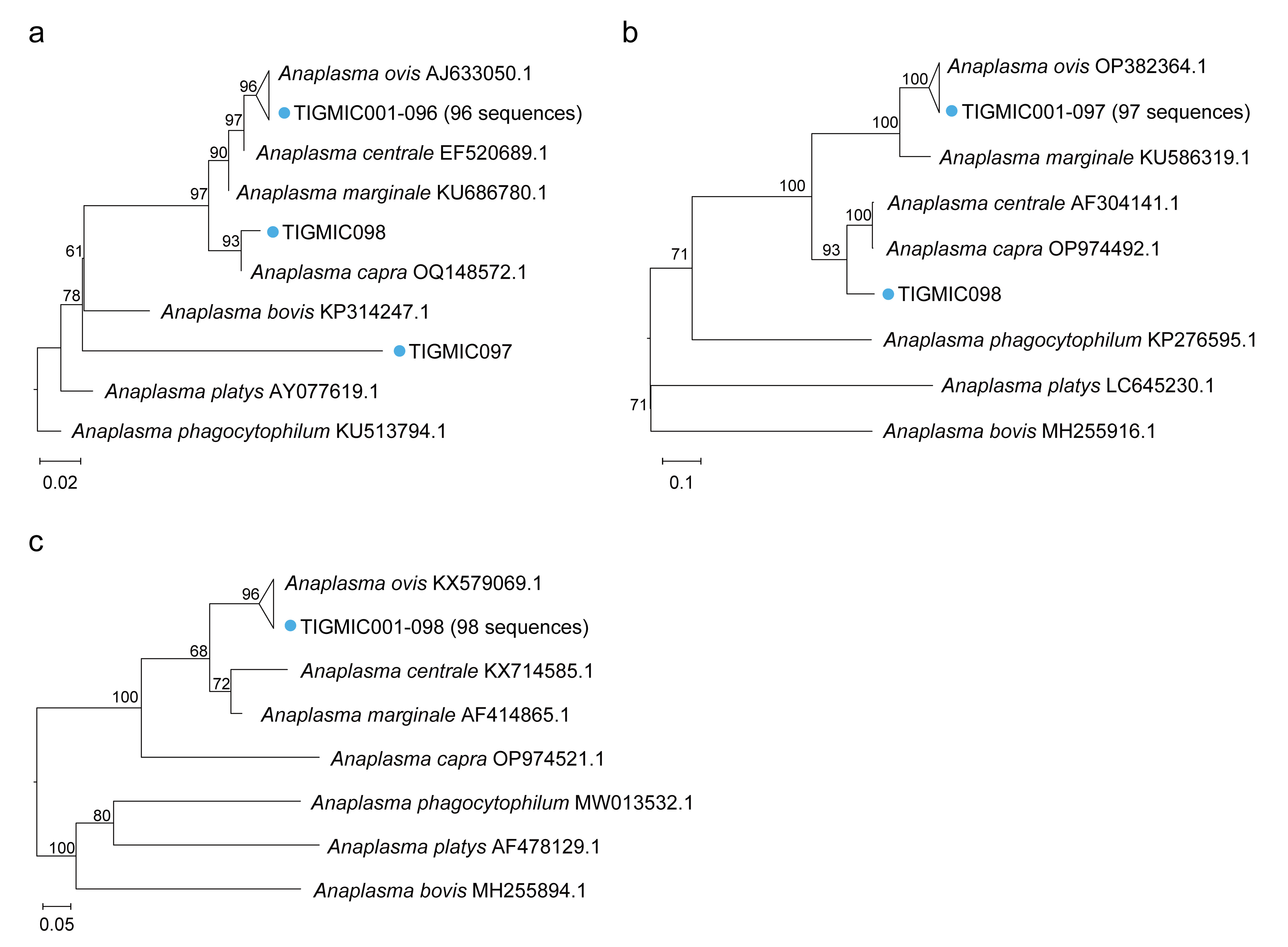

Supplement: S3 Fig — The trees were constructed based on the nucleotide sequences of (a) rrs (660 bp), (b) gltA (793 bp) and (c) groEL (1100 bp) using the maximum-likelihood method with the best substitution model found. All bootstrap support values from 1,000 replicates are shown at the interior branch nodes. The sequences obtained in this study are marked by blue circles. (TIF) [file pntd.0012729.s010.tif]

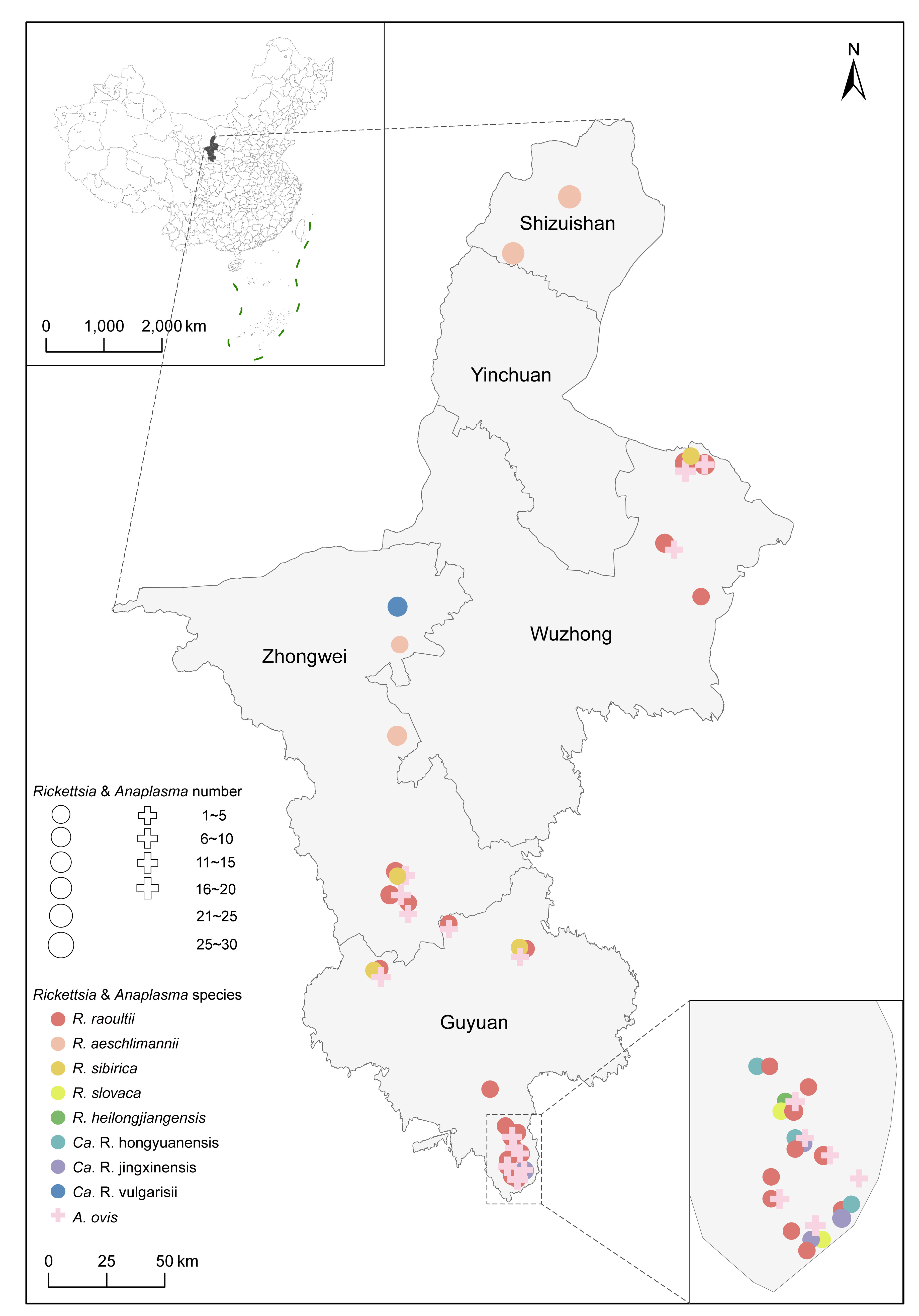

Supplement: S4 Fig — Different colour and size of circles (and cross symbols) represent the species and number of Rickettsia and Anaplasma detected from ticks. The map was constructed using ArcGIS v10.8.2 software. The basemap shapefiles were downloaded from the Chinese Resource and Environmental Science Data Platform (http://www.resdc.cn/, DOI:10.12078/2023010102). (TIF) [file pntd.0012729.s011.tif]
